# Power, false discovery rate and Winner’s Curse in eQTL studies

**DOI:** 10.1093/nar/gky780

**Published:** 2018-09-05

**Authors:** Qin Qin Huang, Scott C Ritchie, Marta Brozynska, Michael Inouye

**Affiliations:** 1 Cambridge Baker Systems Genomics Initiative, Baker Heart and Diabetes Institute, 75 Commercial Rd, Melbourne 3004, Victoria, Australia; 2 Department of Clinical Pathology, University of Melbourne, Parkville 3010, Victoria, Australia; 3 Cambridge Baker Systems Genomics Initiative, Department of Public Health and Primary Care, University of Cambridge, Cambridge CB1 8RN, UK; 4 The Alan Turing Institute, London, UK

## Abstract

Investigation of the genetic architecture of gene expression traits has aided interpretation of disease and trait-associated genetic variants; however, key aspects of expression quantitative trait loci (eQTL) study design and analysis remain understudied. We used extensive, empirically driven simulations to explore eQTL study design and the performance of various analysis strategies. Across multiple testing correction methods, false discoveries of genes with eQTLs (eGenes) were substantially inflated when false discovery rate (FDR) control was applied to all tests and only appropriately controlled using hierarchical procedures. All multiple testing correction procedures had low power and inflated FDR for eGenes whose causal SNPs had small allele frequencies using small sample sizes (e.g. frequency <10% in 100 samples), indicating that even moderately low frequency eQTL SNPs (eSNPs) in these studies are enriched for false discoveries. In scenarios with ≥80% power, the top eSNP was the true simulated eSNP 90% of the time, but substantially less frequently for very common eSNPs (minor allele frequencies >25%). Overestimation of eQTL effect sizes, so-called ‘Winner’s Curse’, was common in low and moderate power settings. To address this, we developed a bootstrap method (BootstrapQTL) that led to more accurate effect size estimation. These insights provide a foundation for future eQTL studies, especially those with sampling constraints and subtly different conditions.

## INTRODUCTION

Genome-wide association studies (GWAS) have identified thousands of genetic variants associated with complex phenotypes (1) and the vast majority of genome-wide significant SNPs are located in non-coding region (2), making interpretation challenging. Integration of gene expression and genetic variation is a ubiquitous approach for uncovering genetic regulatory effects and their ramifications for pathways relevant to human diseases and traits (3–6), and indeed trait-associated SNPs have been found to be enriched for expression quantitative trait loci (eQTL) effects (7).

Yet, while eQTL analysis has become a focus of functional genomics, the lack of a strong evidence base for eQTL study design leaves fundamental questions unanswered. In particular, while more and more eQTLs reach statistical significance, the true proportion of false discoveries and the accuracy of their effect size estimates have not yet been well characterized. A seminal early study compared multiple testing correction methods for detecting eQTLs (including Bonferroni correction, false discovery rate (FDR) control and permutation) using HapMap data; however, estimates of FDR and sensitivity are not possible without knowledge of all true eQTLs in the data (8). Previous eQTL simulations are typically part of new methodologies, yet these simulations have been limited in their reflection of real data. Genotype data have typically been simulated with a narrow minor allele frequency (MAF) range assuming Hardy–Weinberg equilibrium (e.g. MAF 30% in (9), 5 and 20% in (10), 40% in (11)), thus they have not captured realistic patterns of genetic variation, especially linkage disequilibrium (LD) complexity. Furthermore, MAFs at 1% or greater are typically utilized for eQTL analysis (Supplementary Table S1). Others have simulated only a fixed sample size (11–13). Typically, eQTL studies have sample sizes of 50 to 1000, with the accessibility of the tissue or condition a major determining factor (Supplementary Table S1). A recent *trans-*eQTL study performed in whole blood had a size of 5257 samples (6) and a study combined data for 2116 whole blood samples to identify context-specific eQTLs (14). Perhaps the exemplar multiple human tissue resource, the Genotype-Tissue Expression (GTEx) project (15), comprises 44 tissues with a sample size range of 70–361 in its V6p data release (16).

While studies have generally converged on linear regression or linear mixed models for eQTL detection, the multiple testing correction approach is still a source of substantial variability among studies. Various approaches are available for minimizing type I errors. Often criticized as too conservative, particularly with complex LD patterns, the Bonferroni correction aims to control the familywise error rate (the probability of making any type I error) by setting the significance level at *α/N*, where α is the desired significance level (0.05 conventionally) and *N* is the number of tests. FDR-controlling procedures, which aim to control the expected proportion of false discoveries among all rejected null hypotheses, are generally considered to provide a better balance between false positives and false negatives. Benjamini and Hochberg (BH) proposed a procedure (17) assuming each statistical test is independent, which is not the case due to LD. Benjamini and Yekutieli (BY) modified the FDR procedure to one which, while more conservative, accommodates correlation structure between statistical tests (18). The *q*-value FDR-controlling approach from Storey and Tibshirani (ST) estimates the proportion of hypotheses that are truly null (*π*_0_), while the BH procedure assumes *π*_0_ = 1, which makes ST less conservative than the BH procedure (19).

Other approaches have been proposed to deal with multiple testing specifically for eQTL studies. Locus-restricted permutation testing is widely used to obtain empirical null distributions. To achieve this, sample labels are randomly shuffled, while keeping genotype data constant, with association tests performed at each permutation step. For each gene, the best SNP association at each permutation is kept to generate an empirical null distribution of minimum *P*-values, from which permutation test *P*-values are calculated for each *cis-*SNP. Thousands of permutations are required to achieve accurate results, thus there is a high computational cost. Approximations have been investigated for calculating permutation *P*-values, such as those in FastQTL (20) and MVN (21). For example, FastQTL provides an option to approximate the tail of the empirical null distributions of *P*-values using a beta distribution thereby reducing the number of permutations required (20). In addition to permutation tests, eigenMT proposed by Davis *et al.* (22) adjusts *P*-values in shorter time. The number of independent tests (typically SNPs) for each gene is estimated by eigenMT using a genotype correlation matrix, then a Bonferroni procedure is applied (22). Both FastQTL and eigenMT account for LD structure among local variants. Recently, hierarchical procedures, such as TreeQTL (23), have been proposed, which first control for multiple testing of variants at each gene, before controlling for multiple testing across all genes. Taken together, with many correction methods available, it is not clear which method is optimal for eQTL mapping nor what their respective performances are for genetic variants with difference characteristics (allele frequency, effect size etc.).

Effect size estimation for eQTLs represents a more complex and less explored problem, yet its importance is increasing as comparison of eQTLs across tissues, experimental conditions and meta-analyses becomes more common. Furthermore, prediction of tissue-specific gene expression from genotypes, for example using the tool PrediXcan (24), is critically dependent on effect size estimation, particularly *cis*-eQTL effect sizes obtained from analyses of GTEx and other studies. Conversely, a method that predicts genotypes at eQTL SNPs (eSNPs) based on measured gene expression levels has also been proposed (25).

A well-recognized and pervasive phenomenon in GWAS is ‘Winner’s Curse’ (26–29), an ascertainment bias where the true genetic effect is smaller than its estimate within the discovery cohort. Notably, a recent paper from Palmer and Pe’er systematically evaluated summary statistics from 100 previously published quantitative trait studies and showed that Winner’s Curse was a key reason for the non-replicability of significant loci (30). Using a maximum likelihood method, they showed that correction for Winner’s Curse improved replication (30), yet these estimators, based on summary statistics, were shown to over-correct Winner’s Curse and the downward bias was larger when the sample size was small. Palmer and Pe’er definitively established the QTL study-level ramifications of Winner’s Curse, yet to our knowledge no study has comprehensively investigated Winner’s Curse for eQTLs or other QTLs of the expressed genome using individual-level data. To rigorously evaluate each locus and design follow-up experiments, it is important that we understand Winner’s Curse in the context of sample size, allele frequency and the estimated effect size, as well as design methods for adjusting effect sizes during eQTL discovery. As with other studies (31,32) evaluating key genome-wide study design questions, large-scale simulation, where the true causal variant(s) and their effect(s) are known from the outset, is a critical tool for quantifying the relative performance of different approaches in diverse settings.

**Figure 1.**
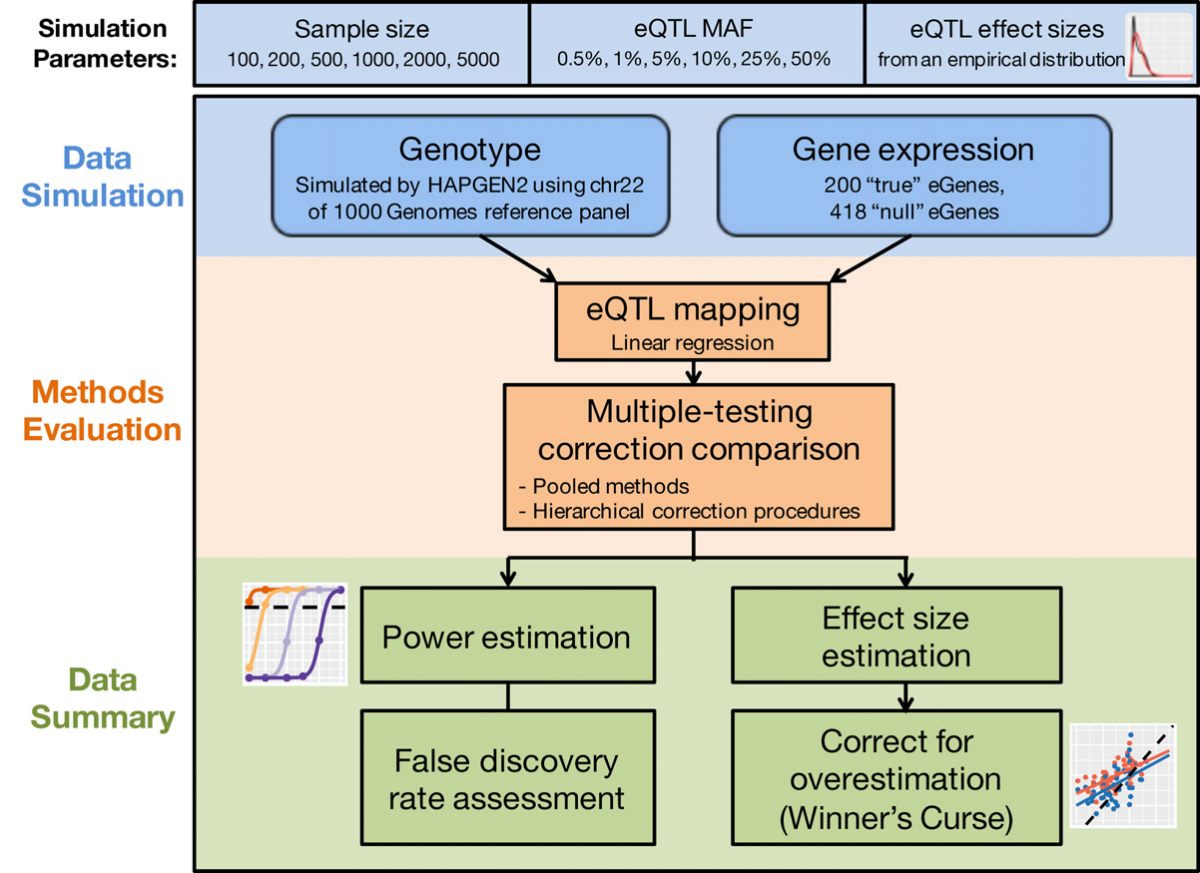
Flowchart of eQTL simulation study.

Here, we used extensive simulations of realistic LD patterns of human genetic variation and matched gene expression to investigate how various scenarios, including different sample sizes, allele frequencies and genetic effect sizes, influence statistical power and FDR (Figure 1). In each scenario, we randomly selected SNPs as true causal *cis*-eQTLs, each associated with expression levels of a target gene. We performed eQTL mapping and evaluated a variety of multiple testing correction methods, used both individually and hierarchically, under each scenario. We next investigated the accuracy of genetic effect size estimation across scenarios, the effect of the Winner’s Curse, and how bias was affected by study power. At last, we evaluated the accuracy of a variety of eQTL effect size estimation procedures.

## MATERIALS AND METHODS

### Simulating genotypes and selecting eQTLs

Genotype data were simulated using HAPGEN2 (33) based on the 99 FIN haplotypes of chromosome 22 from the 1000 Genomes Project data (phase3, GRCh37) (34). The simulated genotypes had similar LD patterns with the reference data. Six sets of genotype data were generated at varying sample sizes: 100, 200, 500, 1000, 2000 and 5000 individuals. After filtering out SNPs with MAF <0.5% or Hardy–Weinberg equilibrium *P*-value <5 $ \times$ 10^−6^, ∼150 thousand SNPs remained in each data set.

We explored six different true eSNP MAFs (0.5, 1, 5, 10, 25 and 50%) in each of the six genotype datasets, resulting in 36 scenarios in total. In each scenario, 200 SNPs at the scenario MAF were randomly chosen as true causal eSNPs, each regulating the expression of a randomly selected *cis* gene (within ±1 Mb from transcription start site (TSS) of the gene). These 200 causal eSNPs were selected from an LD pruned subset where the pairwise *r*^2^ was ≤0.3.

### Simulating gene expression

To get a distribution of *cis*-eQTL effect sizes, we first performed eQTL mapping in DILGOM dataset (35,36) using additive linear model with covariates that accounted for gender, age and population structure. Expression data were further scaled to make each gene’s expression across samples follow a standard normal distribution. To avoid an inflated number of associations due to LD structure among variants, we kept only the best association with the minimum nominal *P*-value for each gene. As shown in simulation results, only eQTLs with large effect sizes could be identified given a limited sample size. To reduce the bias caused by limited power, we included all genes to obtain the effect size distribution and fit it with a gamma distribution, from which we randomly selected true effect sizes.

First, we performed a set of simulations in which the expression of 200 genes were simulated, each regulated by a single causal eSNP, varying the study sample size, as well as the MAF and effect size of the causal eSNP. In each scenario, 200 genes out of 618 genes on chromosome 22 were designated as ‘true eGenes’ regulated by a causal eSNP each and the remaining 418 as ‘null genes’ with no truly associated eSNPs. The 200 true associations were modelled by a simple linear regression:

\begin{equation*}{{{y}}_{{i}}} = {{\beta }}{{{g}}_{{i}}} + {{{\varepsilon }}_{{i}}}\,{\rm{with}}\,{{{\varepsilon }}_{{i}}}\sim{{N}}\left( {0,\,1} \right),\end{equation*}

where *y_i_* denoted the expression level of an eGene for individual *i, β* the genetic effect size of the corresponding eSNP, *g_i_* the minor allele dosage of the eSNP coded as 0, 1 or 2, and *ϵ_i_* the error variance for the *i*th individual, which followed a standard normal distribution. For 418 null genes, no genetic effects were simulated (*β* = 0) and the simulated expression was normally distributed. True eGenes effect sizes were randomly drawn from a gamma distribution derived from a real dataset as described above. In scenarios where causal eSNPs had a constant effect size, *β* was 0.25, 0.5, 1 or 1.5.

Additional simulations were performed to examine the consequences of the following for multiple testing correction: the assumption of error normality in the simulations, correlation structure amongst gene expression, non-linear eSNP effects and multiple causal eSNPs. In all simulations, including those above, 100 replicates were performed to obtain estimates of sensitivity and FDR under each scenario for each multiple testing correction method described in the next section below.

To examine the assumption of error normality, we simulated gene expression as described above, but changing the error term to be drawn from a log-normal distribution (with mean and s.d. of the variable’s natural logarithm 0 and 1, respectively). Simulations were additionally performed in which gene expression profiles were inverse rank normalized across samples using the ‘rntransform’ function in the GenABEL R package (37).

To examine the effect of gene coexpression on eQTL mapping, we simulated correlated expression amongst adjacent genes arising from a single shared causal eSNP. Chromosome 22 was divided into 35 genomic blocks with a length of 1 Mb. Two hundred true eGenes were randomly selected from all 618 genes, and true eGenes within each genomic block were simulated to have correlated gene expression levels, sharing the same causal eSNP. Correlated expression *y*_1_, *y*_2_, …, *y_i_* for each true eGene*_1_*, eGene*_2_*, …, eGene*_i_* in block *j* were simulated as following:

\begin{equation*}\begin{array}{@{}*{1}{c}@{}} {\ {y_1} = {\beta _j}\ \times {g_j} + {\varepsilon _1},}\\ {\ {y_2} = {\beta _j}\ \times {g_j} + {r_2} \times {\varepsilon _1} + \sqrt {1 - {r_2}^2} \times {\varepsilon _2},}\\ { \ldots ,}\\ {\ {y_i} = {\beta _j}\ \times {g_j} + {r_i} \times {\varepsilon _1} + \sqrt {1 - {r_i}^2} \times {\varepsilon _i}.} \end{array}\end{equation*}

All *i* true eGenes in this block shared a causal eSNP*_j_*, which was coded as (0, 1, 2), and had the same genetically regulated component (${\beta _j} \times {g_j}$) where ${\beta _j}$ was the effect size of the SNP on each true eGene. Error terms (${\varepsilon _1}$, ${\varepsilon _2}$, …, ${\varepsilon _i}$) followed a standard normal distribution. For each eGene*_i_*, except for the first eGene*_1_*, the noise component (${r_i} \times {\varepsilon _1} + \sqrt {1 - {r_i}^2} \times {\varepsilon _i}$) followed a standard normal distribution that was correlated with the error term of the first eGene_1_ (${\varepsilon _1}$) with a correlation coefficient ${r_i}$, which was randomly drawn from a uniform distribution *U*(0.6, 0.9).

To examine the effect of non-linear eSNPs on multiple testing correction, two additional simulations were performed. One in which all causal eSNPs had dominant effects, and the other in which all causal eSNPs had recessive effects. To simulate dominant effects, causal eSNPs were coded as (0, 2, 2) based on the absence/presence of one or more copy of the minor allele. Conversely, to simulate recessive effects, causal eSNPs were coded as (0, 0, 2). Apart from the causal eSNP coding, simulations were as described at the beginning of this section, where 200 true eGenes were randomly selected and simulated to have a single causal eSNP with a standard normal error term.

To examine the effect of multiple causal eSNPs on multiple testing correction, two additional simulations were performed. One in which each true eGene was regulated by two causal eSNPs, and one in which each true eGene was regulated by three causal eSNPs, with additive effects on gene expression as follows:

Two causal eSNPs:

\begin{equation*}y\ = {\beta _1}\ \times {g_1} + {\beta _2} \times {g_2} + \varepsilon ,\end{equation*}

Three causal eSNPs:

\begin{equation*}y\ = {\beta _1}\ \times {g_1} + {\beta _2} \times {g_2} + {\beta _3} \times {g_3} + \varepsilon ,\end{equation*}

where effect sizes ${\beta _1}$, ${\beta _2}$ and $\ {\beta _3}$ were drawn from the gamma distribution described at the start of the section based on the distribution of effect sizes observed in a real dataset. The terms ${g_1}$, ${g_2}$ and ${g_3}$ describe the minor allele dosage of the each causal eSNP, respectively. In each simulation, the first causal eSNP was randomly selected as described above, based on the desired MAF for each scenario. Additional causal eSNPs at each eGene were randomly selected from nearby variants in LD with ${g_1}$ based on the distribution of LD correlation observed between multiple causal eSNPs observed in a conditional eQTL study of ∼5000 peripheral blood samples (38), following a beta distribution with the shape parameters 2.6 and 4.5. Using this selection scheme, MAFs tended to be similar across the multiple causal eSNPs at each eGene (Supplementary Figure S20). The error term $\varepsilon$ was drawn from a standard normal distribution as described above.

### Mapping eQTLs and correcting for multiple testing

For *cis*-eQTL analysis, we used Matrix eQTL (39) to fit linear regression models between each gene and the minor allele dosage of all SNPs located within 1 Mb of the gene's TSS. To adjust for multiple tests, we applied either (i) a correction method to all hypotheses (pooled method) or (ii) a hierarchical correction procedure, where two methods were used in combination to correct for multiple SNPs tested for each gene and multiple genes separately.

Pooled multiple testing correction was performed using either Bonferroni correction or FDR-controlling procedures applied to all SNP–gene hypothesis tests. Bonferroni correction (pooled Bonferroni), Benjamini and Hochberg (17) (pooled BH) and Benjamini and Yekutieli (18) (pooled BY) FDR procedures were performed using ‘p.adjust’ function in R (40), and Storey and Tibshirani (19) (pooled ST) procedure was performed by the R package ‘qvalue’ (41).

A three-step procedure was employed to perform hierarchical multiple testing correction. In Step 1, *P*-values of all *cis*-SNPs were adjusted for multiple testing for each gene separately (locally adjusted *P*-value). In Step 2, the minimum adjusted *P*-value from Step 1 was taken for each gene, then these adjusted *P*-values were further adjusted for multiple testing across all genes (globally adjusted *P*-value). At last, in Step 3, significant eSNPs were identified for each significant eGene as SNPs with a locally adjusted *P*-value from Step 1 lower than the locally adjusted minimum *P*-value corresponding to the globally adjusted *P*-value threshold of 0.05.

Hierarchical multiple testing correction was performed using different combinations of multiple testing correction methods in Step 1 and Step 2 described above. In Step 1, we applied FDR procedures (ST, BH or BY), Bonferroni, eigenMT (22) or permutation approaches to correct for multiple local SNPs tested for each gene. In Step 2, we applied three FDR-controlling procedures or Bonferroni correction to control the rate of false positive eGenes. Note that eigenMT and permutation approaches are used hierarchically by design.

When Bonferroni was used as a local correction method, the adjusted *P*-value was calculated by multiplying each linear model *P*-value by the number of SNPs in the corresponding 1 Mb *cis* window for the tested gene. When using eigenMT, the linear model *P*-value was multiplied by the number of effective independent tests estimated from the genotype correlation matrix by eigenMT (in Python 2.7.3) (22). Permutations were performed by shuffling sample labels of expression data. For each gene, minimum nominal *P*-values from all permutation tests were kept to obtain the null distribution. Permutation *P*-values were calculated as the proportion of permutations showing more significant minimum *P*-value than the observed nominal *P*-value. The null distribution used to calculate permutation *P*-values was either (i) the exact distribution from permutations (exact permutation scheme) or (ii) a beta distribution approximation of the null distribution tail, which is implemented in FastQTL (version 2.0) (20). When using FastQTL, we performed either a fixed number of permutations (1000) or under an adaptive scheme, a number ranging from 100 to 10 000 permutations determined via iterative estimates of gene significance throughout the permutation procedure.

When calculating the sensitivity and FDR of multiple testing correction methods, true positives and false discoveries were calculated at the gene level. If any significant SNPs were in high LD (*r*^2^ ≥ 0.8) with any simulated causal eSNP, an eGene was considered a true positive. Conversely, if there were significant SNPs for an eGene but it was not simulated to be a true eGene or no significant SNPs were in high LD (*r*^2^ ≥ 0.8) with any simulated causal eSNP, it was considered a false discovery.

### Conditional analyses

In simulations of multiple causal eSNPs, a two-stage conditional analysis (42) was performed to identify independent eSNPs for each significant eGene after eQTL mapping and hierarchical multiple testing correction with eigenMT-BH. The nominal *P*-value threshold corresponding to the global correction FDR 0.05 cut-off calculated via eigenMT-BH in the initial eQTL scan was used to determine significance in the conditional analysis.

The conditional analysis comprised two stages: a forward stage and a backward stage. The forward stage consisted of an iterative procedure. At each iteration, *cis*-eQTL mapping was performed for each significant eGene, adjusting for the top SNP identified in the initial eQTL mapping. If any SNPs remained significant after adjusting for this top SNP, the new top SNP was added to the list of independent eQTL signals and adjusted for in subsequent iterations. If no SNPs were significant, the iterative procedure terminated and proceeded to the backward stage. In the backward stage, each independent eQTL signal was tested separately using a leave-out-one model adjusting for all other SNPs in the list of independent eQTL signals as covariates. The final set of independent eQTLs comprised the set of eSNPs that remained significant in the backward stage.

When calculating the sensitivity and FDR of the conditional analyses, each independent eQTL signal was considered a true positive if it was in high LD (*r*^2^ ≥ 0.8) with any simulated causal eSNP. Conversely, an independent eQTL signal was considered a false discovery if it was not in high LD with any simulated causal eSNP. Where two or more independent eQTL signals were identified and in high LD with any causal eSNP, only one signal was considered a true positive while all others were considered false discoveries.

### Correcting for Winner’s Curse

To evaluate and correct the effect of the Winner’s Curse, we considered the effect size estimates of the SNP with the minimum *P*-value (top eSNP) for each eGene. We use ${\hat{\beta }_{N( e )}}$ to denote the ‘naïve estimator’: the beta coefficient obtained from the linear regression of each eGene on its top eSNP.

We adjusted a bootstrap method (43) to re-estimate eQTL effect sizes of significant eGenes determined by a hierarchical correction procedure (Bonferroni-BH by default; eigenMT-BH is also recommended). This approach consists of a repeated bootstrap analysis, in which random samples are drawn with replacement from the study dataset to partition the study samples into two groups: a bootstrap detection group of identical size to the original dataset comprising samples randomly selected with replacement and a bootstrap estimation group comprising the remainder of the study samples. Due to the sampling with replacement, the bootstrap detection group typically comprised 63.2% of the study samples while the bootstrap estimation group comprised the other 36.8% of samples. The effect size is then estimated separately in the bootstrap detection and estimation groups for each eGenes and its top eSNP based on the original dataset.

After performing the above procedure with 200 bootstraps, three bootstrap estimators were calculated and compared for eGene effect size re-estimation:

a shrinkage estimator:

\begin{equation*}{\hat{\beta }_{N\left( e \right)}} - \frac{1}{{{B_{\left( e \right)}}}}\mathop \sum \nolimits_{i\ = \ 1}^{{B_{\left( e \right)}}} \left( {{{\hat{\beta }}_{D\left( e \right)i}} - {{\hat{\beta }}_{E\left( e \right)i}}} \right);\end{equation*}

an out-of-sample estimator:

\begin{equation*}\frac{1}{{{B_{\left( e \right)}}}}\mathop \sum \nolimits_{i\ = \ 1}^{{B_{\left( e \right)}}} {\hat{\beta }_{E\left( e \right)i}};\end{equation*}

and a weighted estimator:

\begin{equation*}\left( {1 - \omega } \right){\hat{\beta }_{N\left( e \right)}} + \omega \frac{1}{{{B_{\left( e \right)}}}}\mathop \sum \nolimits_{i\ = \ 1}^{{B_{\left( e \right)}}} {\hat{\beta }_{E\left( e \right)i}}.\end{equation*}

Where ${\hat{\beta }_{D( e )i}}$ denotes the effect size of eGene *e* in each bootstrap detection group *i*, ${\hat{\beta }_{E( e )i}}$ denotes the effect size of eGene *e* in each bootstrap estimation group *i* and ${B_{( e )}}$ denotes the number of bootstraps in which the association between the eGene *e* and its top eSNP was significant in the bootstrap detection group (thus ${B_{( e )}}$ ≤ 200). An association between an eGene and its top eSNP was considered significant in the bootstrap detection group if its locally adjusted *P*-value (corrected for multiple *cis*-SNPs within 1 Mb of the respective eGene using e.g. eigenMT or Bonferroni) was smaller than the locally adjusted *P*-value corresponding to the 0.05 threshold after global adjustment (e.g. BH) in the eGene detection analysis prior to performing the bootstrap procedure. For the weighted estimator, the weight *w* was 0.632, i.e. the proportion of unique samples in the bootstrap detection group.

## RESULTS

### Simulation of *cis*-eQTL data

To assess the power, FDR and effect size estimation of eQTL studies based on different parameters, we simulated 36 scenarios with combinations of six sample sizes (*N* = 100, 200, 500, 1000, 2000 and 5000) and six true minor allele frequencies (MAFs) of eSNPs (MAF = 0.5, 1, 5, 10, 25 and 50%). Realistic LD patterns were simulated using HAPGEN2 (33) with chromosome 22 of the 1000 Genomes Project phase 3 data (34) as reference. In each scenario, 618 gene expression traits were simulated, among which 200 were under genetic regulation (true eGenes). Each true eGene was simulated to be regulated by one *cis*-eQTL with a genetic effect size randomly drawn from an empirical distribution based on eQTL analysis of a real dataset (35,36).

For each gene, all SNPs located within 1Mb of the TSS were tested for association using linear regression models through Matrix eQTL (39). We mapped *cis-*eQTLs for the 36 scenarios separately and evaluated different multiple testing correction methods. Figure 1 illustrates the workflow of our eQTL simulations and methods evaluation. We used Bonferroni, FDR-controlling procedures, permutation approaches and eigenMT to correct for multiple testing. The Bonferroni and FDR procedures were applied alone to all hypotheses (pooled method) and were also used in combination via a hierarchical correction procedure (‘Materials and Methods’ section). We repeated the simulation for each scenario 100 times and calculated the sensitivity and FDR of each multiple testing correction method based on all simulations.

### Power and false discovery rate between scenarios and multiple testing correction procedures

We first assessed the variability in sensitivity and FDR for the various multiple testing correction methods for eGene detection across simulation scenarios. A significant eGene was considered a true positive if: (i) it was among the 200 true eGenes simulated, and (ii) the simulated causal eSNP for that eGene was among the significant eSNPs, or a significant eSNP was in high LD with the causal eSNP (*r*^2^ ≥ 0.8). For each multiple testing correction method, sensitivity, or true positive rate (TPR), was calculated as the proportion of simulated true eGenes correctly identified as true positives. Conversely, the FDR was calculated as the proportion of false positives in significant eGenes identified across all 100 simulations.

We evaluated multiple testing correction methods in two ways: first, applied across all SNP–gene hypothesis tests (hereby ‘pooled methods’) and second, in combinations in a hierarchical approach in which SNP–gene hypothesis tests were partitioned into groups by the gene being tested (hereby ‘hierarchical correction procedures’) (44). In the case of hierarchical correction procedures, the multiple hypothesis tests of eGenes were controlled (Step 2, global correction) based on the multiple testing adjusted statistics (Step 1, local correction) of each gene’s best association, then SNPs significantly associated with the significant eGenes were identified based on the locally corrected *P*-value corresponding to the threshold of 0.05 after global correction (Step 3, ‘Materials and Methods’ section).

FDR-controlling procedures applied to all hypotheses (pooled FDR methods) failed to control the FDR of eGenes in nearly all scenarios (Supplementary Figure S1). We applied three FDR-controlling procedures to all hypotheses: the Storey and Tibshirani (ST) (19), Benjamini and Hochberg (BH) (17) and Benjamini and Yekutieli (BY) (18) procedures. The ST and BH procedures failed to control FDR at the desired level of 0.05 in majority of the scenarios, and FDR increased with sample size, reaching more than 0.6 under scenarios with sample sizes of 2000 or 5000 and true eSNP MAFs ≥25% (Supplementary Figure S1A). The BY procedure was the most conservative method among pooled FDR procedures but still had inflated FDR under scenarios with large sample sizes (≥1000) and true eSNP MAFs ≥25%. As expected, a pooled Bonferroni correction had very low FDR values in most scenarios, with the lowest sensitivity across MAFs and sample sizes (Supplementary Figure S1). However, even pooled Bonferroni correction failed to control FDR of rare variant eQTLs (MAF ≤1%) in scenarios with <1000 samples. Overall, we observed inflated rates of false positive eGenes for all pooled FDR methods.

**Figure 2.**
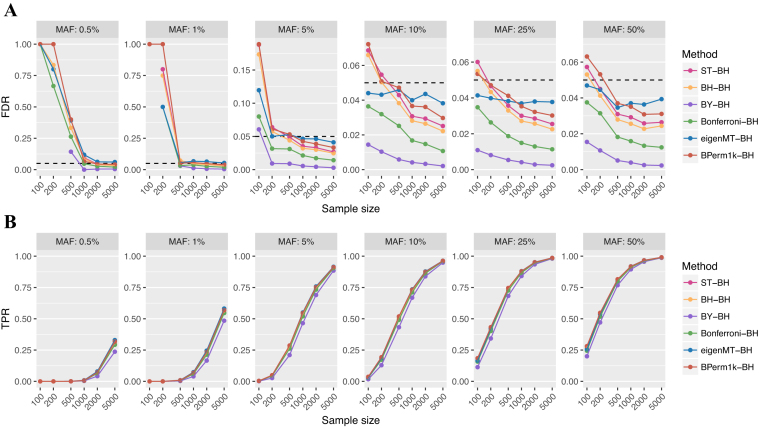
FDR and sensitivity of selected hierarchical multiple testing correction methods. Comparison of the FDR (**A**) and sensitivity/TPR (**B**) of six methods (different colours) for controlling multiple testing of SNPs at each gene (local correction), with BH used to control for multiple testing across all genes (global correction). The six methods compared were Storey and Tibshirani (ST), Benjamini and Hochberg (BH), Benjamini and Yekutieli (BY), Bonferroni correction, eigenMT and permutation tests based on beta approximation (BPerm1k). Comparison of all combinations of multiple testing correction methods for hierarchical correction is shown in Supplementary Figures S2 and S3. Application of BH in the global correction step had the best sensitivity for all methods used in the local correction step of any hierarchical correction procedures. Each dot represents one scenario and plots show different MAFs of the simulated causal eSNPs. The dashed horizontal lines in panel (A) indicate the desired FDR level of 5%. Scenarios where no significant eGenes were identified are not shown in panel (A).

In contrast to pooled methods, we observed better calibrated FDR for hierarchical multiple testing correction procedures, except in scenarios with low statistical power (Figure 2A and Supplementary Figure S2). We compared ST, BH, BY, Bonferroni, eigenMT and three permutation approaches (discussed in a later paragraph) for adjusting the *cis*-SNP *P*-values for each simulated gene (local correction), combined with a comparison of the ST, BH, BY and Bonferroni correction for adjusting the subsequent minimum adjusted *P*-value across all genes (global correction).

We observed lower sensitivity as well as lower FDR than ST and BH when applying BY and Bonferroni to correct across genes, regardless of which multiple testing correction method was used for local correction (Supplementary Figures S2 and S3). ST and BH global correction had identical performance, except when permutation tests were used as local correction method, where ST had higher FDR than BH and often had FDR slightly higher than 5% (Supplementary Figures S2 and S3). We therefore subsequently focused on the BH procedure to control for multiple testing across genes in hierarchical correction procedures.

We compared three different permutation approaches to correct for multiple testing at each gene: (i) using exact permutation test *P*-values from 1000 permutations (Perm1k-BH), (ii) using *P*-values obtained from beta distribution approximation of each null distribution’s tail after 1000 permutations (BPerm1k-BH) and (iii) using beta approximation under an adaptive scheme where a minimum of 100 and a maximum of 10 000 permutations were performed for each gene based on the significance level of this gene (APerm10k-BH). Due to the prohibitive computational time required to run Perm1k and APerm10k, we ran 10 simulations rather than 100 to compare the three permutation approaches. Perm1k-BH had lower sensitivity than the other two permutation approaches in scenarios with low detection power and it also had a higher FDR (Supplementary Figure S4). BPerm1k and APerm10k had similar performance, indicating 1000 permutations were sufficient to obtain an accurate approximation of the *P*-value null distribution tail. We therefore used BPerm1k-BH as a representative of permutation approaches to compare with other multiple testing correction methods.

Amongst the hierarchical correction methods with BH as global correction, BY adjustment of multiple SNPs (BY-BH) had the most conservative FDR among all methods, more so than Bonferroni-BH due to BY’s heavier correction for the lowest *P*-values; however, this came at the expense of lower sensitivity (Figure 2). Besides BY-BH, other methods did not show a notable difference in sensitivity. Perhaps surprisingly, Bonferroni-BH maintained a comparable sensitivity to other methods while having an FDR well below 0.05. In terms of calibration, eigenMT-BH had an FDR closest to 0.05 and was relatively stable with respect to sample size, whereas other methods showed an inverse relationship between FDR and sample size. In the ‘Discussion’ section, we explore the trade-offs of FDR calibration versus minimization for a given power. Below, we utilize the eigenMT-BH procedure to illustrate the ramification of our findings for eQTL study design, while also noting that design differences between Bonferroni-BH and eigenMT-BH would be minor.

These observations were robust under a variety of more complex simulations. Relative performance of hierarchical multiple testing procedures in terms of FDR calibration and sensitivity remained the same when simulating (i) log-normal noise (‘Materials and Methods’ section ; Supplementary Figure S5), (ii) correlated expression via a shared causal *cis*-SNP (‘Materials and Methods’ section; Supplementary Figure S6), (iii) dominant and recessive causal SNPs (‘Materials and Methods’ section; Supplementary Figures S7 and S8) and (iv) multiple causal cis-SNPs per eGene (‘Materials and Methods’ section; Supplementary Figure S9). However, there were notable scenarios where FDR was inflated above 5%. Simulations of log-normal noise without inverse normal transformation resulted in FDR approaching 1.0 due to pervasive outliers, produced by extreme noise that coincided with low MAF variants (Supplementary Figure S10). Simulations of correlated gene expression (Supplementary Figure S6) showed reduced FDR control at low power across all methods compared to uncorrelated gene expression.

**Figure 3.**
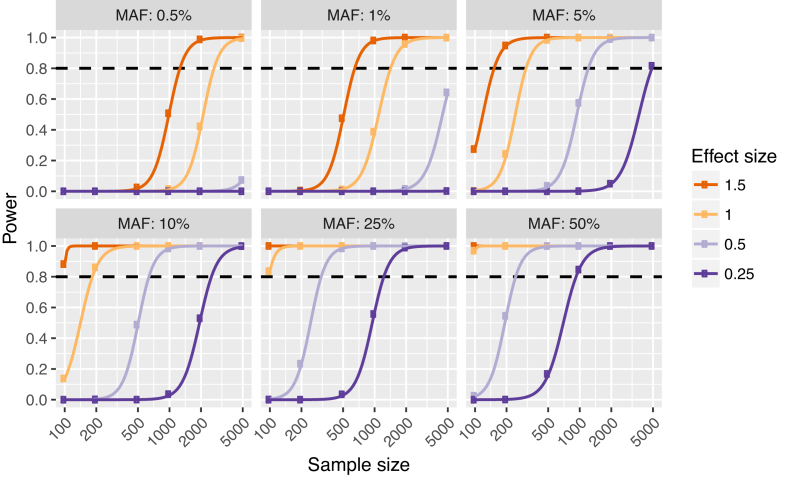
Power and eQTL effect size. A constant genetic effect size (0.25, 0.5, 1.0 or 1.5 s.d. gene expression per allele) was simulated in each scenario. Plots represents different MAFs of the simulated true eSNPs. Sample size increases from left to right on *x*-axes. The estimated statistical power for eGene detection from 100 simulations is shown on *y*-axes. A hierarchical correction procedure using eigenMT for local correction and BH for global correction (eigenMT-BH) was used to correct for multiple testing. The dashed horizontal lines indicate sufficient statistical power (0.8).

Across all effect sizes and using the eigenMT-BH procedure (Figure 2), it was apparent that (i) eSNPs with ≤0.5% and ≤1% MAF that were detected with <1000 and <500 samples, respectively, were likely to be false discoveries, (ii) for studies with 100 samples, a MAF threshold of 10% is necessary to control FDR at ≤5% irrespective of hierarchical multiple testing procedure. Recessive eSNPs detected with standard eQTL analyses (i.e. using linear models) were largely false discoveries when MAF was ≤25% in 100 samples or MAF ≤10% in up to 1000 samples (Supplementary Figure S8). In varying the eSNP effect size (0.25, 0.5, 1.0 or 1.5 s.d. gene expression per allele), we found that sample sizes up to 200 (quite common in the eQTL literature) only reached 80% power for eQTLs of ≥5% MAF and effect size 1.5 s.d. per allele or for eQTLs of 50% MAF and effect size of approximately ≥0.6 s.d. per allele ( Figure 3). The maximum sample size of 5000 in our simulations still did not reach 80% power to detect eQTLs with effect size of 0.25 s.d. per allele and <5% MAF. When sample sizes were >1000 and MAF >25%, eQTLs with effect size of 0.25 s.d. per allele could be detected at power 80%. Studies of 100 samples were underpowered unless eQTLs were moderately common (at least ∼25% MAF) and of large effect size (≥1.0 s.d. per allele).

### Identification of the simulated causal eSNP

When hierarchical multiple testing correction procedures had calibrated FDR for eGenes, we observed multiple significant eSNPs at each true positive eGene (Supplementary Figure S11) despite simulating only one causal eSNP for each true eGene, as would be expected given LD. The number of SNPs significantly associated with a true eGene increased with both sample size and true eSNP MAF, with >1000 significant eSNPs identified per eGene on average in the scenario with the largest sample size (*N* = 5000), true eSNP MAF (50%) and eQTL effect size (1.5 s.d. per allele) (Supplementary Figure S11).

**Figure 4.**
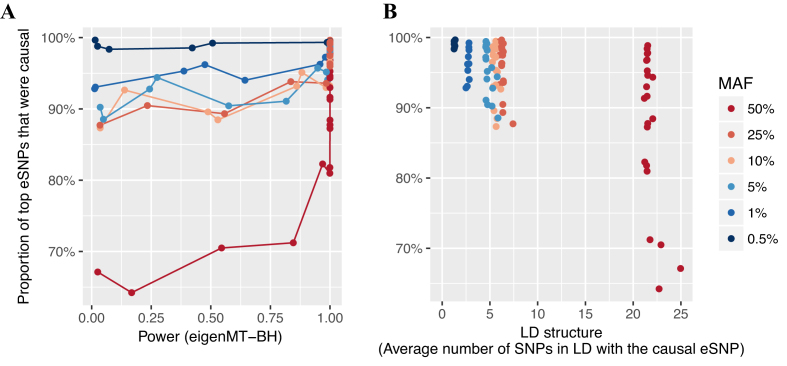
Identification of true causal eSNPs. In each scenario, the 200 causal eSNPs have the same effect size in addition to MAF. For significant true eGenes, the proportion of top eSNPs (minimum *P*-value) that were true causal eSNPs (or in perfect LD) is shown (*y*-axes) for either (**A**) the power to detect eQTLs of the scenario or (**B**) the amount of LD for true causal eSNPs, i.e. the average number of SNPs within 1Mb and in moderate LD (*r*^2^ ≥ 0.5) with the causal eSNP. Scenarios are coloured according to true eSNP MAF. Only scenarios with power ≥0.01 are shown. A hierarchical correction procedure using eigenMT for local correction and BH for global correction (eigenMT-BH) was used to identify eGenes.

Many studies focus on the eSNP with the strongest association (lowest *P*-value) with each eGene (top eSNP) when performing downstream analyses, such as enrichment analysis or effect size estimation (14,16). In our simulations, we found that while the power to detect the presence of an eQTL increased with increasing MAF, the probability that the true causal eSNP was the top eSNP declined (Figure 4A). However, holding MAF constant and increasing study power (increasing sample size and effect size) resulted in increasing probability to detect the true causal eSNP (Figure 4A). In scenarios with at least 1% power to detect an eQTL, top eSNPs with MAF 0.5% were nearly always the true causal eSNP. Given the critical role of LD in fine-mapping, we confirmed our observations were due to a positive relationship between an eSNPs’ MAF and the amount of local LD (Figure 4B). For top eSNPs that were not true causal eSNPs, 83% were in high LD (*r*^2^ ≥ 0.8) with the true causal eSNPs (Supplementary Figure S12). Overall, for studies with 80% power to detect a given eQTL of MAF ≤25%, the top eSNP was the true causal eSNP 90% of the time.

We next investigated the sensitivity and FDR of typical conditional analyses to identify and distinguish between multiple causal eQTL signals, using the nominal eSNP significance *P*-value threshold determined by eigenMT-BH correction (‘Materials and Methods’ section). FDR among independent eQTL signals identified by conditional analyses decreased as sample size increased (Supplementary Figure S13). FDR was slightly inflated when multiple causal eSNPs had MAFs of 50% (Supplementary Figure S13), consistent with the inflated FDR observed in the initial eQTL scan (Supplementary Figure S9), because of the presence of negatively correlated minor allele dosages between the causal eSNPs of an eGene, which was more often observed when causal eSNPs had MAFs of 50% (Supplementary Figure S14). In scenarios where MAFs of causal eSNPs were ≥25%, conditional analyses identified additional causal eSNPs that were not significant in the initial eQTL mapping step (Supplementary Figure S15). Among the top SNPs (at each independent locus) ≥80% were the causal eSNPs (or in perfect LD) when causal eSNPs had MAF of ≤25% (Supplementary Figure S16). The proportion was lower when MAF of causal eSNPs were 50%, consistent with scenarios with a single simulated causal eSNPs (Figure 4A).

### Winner’s Curse in eQTL effect size estimation

**Figure 5.**
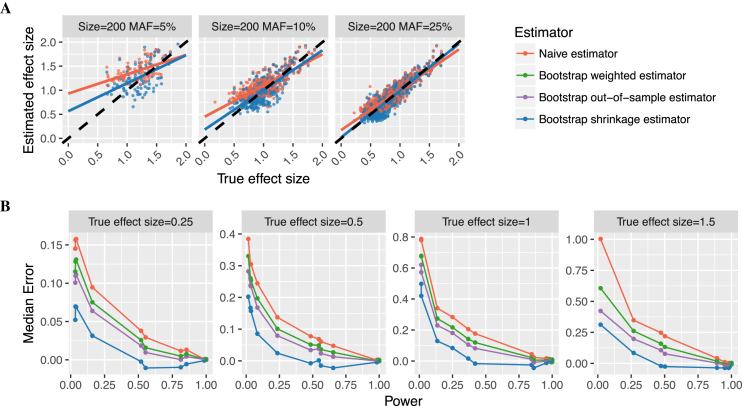
Winner’s Curse in eQTL effect size estimation and correction by bootstrap method. Panel (**A**) shows the phenomenon of Winner’s Curse by three examples: scenarios where the sample size is 200 and the MAFs of causal eSNPs are 5, 10 and 25%. Each dot represents one true positive eGene from 10 simulations of the scenario. Plots compare the estimated effect size (*y*-axes) of the top SNP of each true positive eGene to the true effect size (*x*-axes) of the simulated causal eSNP. Red points show the naïve estimator (beta coefficient from liner regression) and blue points show the bootstrap shrinkage estimator, which was the best estimator (see panel (B)). Red (or blue) lines are linear regression fit of the naïve estimator (or the bootstrap estimator) on the simulated effect size for the true positive eGenes. Black dashed lines in panel (A) indicate where the estimated effect size equals to the true value. Panel (**B**) shows the median error (difference between estimated and true effect size) for all estimators across 10 simulations of scenarios where a constant true effect size (0.25, 0.5, 1 or 1.5 s.d. gene expression per allele) was simulated. A hierarchical correction procedure using eigenMT for local correction and BH for global correction (eigenMT-BH) was used to correct for multiple testing.

To systematically evaluate the effect of Winner’s Curse in eQTL studies, we compared beta coefficients obtained from the Matrix eQTL linear regression models for the top eSNP of each true positive eGene (the ‘naïve estimator’) to their simulated true effect sizes. We observed that median error of the naïve estimator increased as study power decreased, as expected, and also that the naïve estimator consistently overestimated the true effect size with overestimation increasing as power to detect an eQTL decreased (Figure 5 and Supplementary Figure S17).

To address this, we investigated various methods for re-estimating effect sizes. Methods have been proposed to correct for Winner’s Curse in GWAS (27,45), but to our knowledge, no method has yet been designed for bias correction in eQTL studies. We adapted a bootstrap method (43) for eQTL studies and compared three bootstrap estimators (a shrinkage estimator, an out-of-sample estimator and a weighted estimator, see ‘Materials and Methods’ section) to determine the best approach for adjusting for Winner’s Curse. All three bootstrap estimators had more accurate effect size estimates (smaller mean squared error and median error closer to 0) than the naïve estimator when power of eQTL detection was low to moderate (Figure 5B and Supplementary Figure S18). Amongst the three bootstrap estimators, the shrinkage estimator was closest to the true effect size overall and across all study powers. In scenarios with high power for eQTL detection, Winner’s Curse was not apparent, and the bootstrap shrinkage estimator and naïve estimator had similar estimates (Supplementary Figure S19). The bootstrap method for eQTL studies is freely available at https://github.com/InouyeLab/BootstrapQTL.

## DISCUSSION

In this study, we have utilized extensive, realistic simulations of eQTL data to investigate fundamental questions in eQTL study design relating to power, FDR and effect size estimation. The most commonly used MAF cut-offs in recent eQTL studies are 1 or 5% (Supplementary Table S1). For instance, GTEx restricted the association tests to SNPs with minor allele count ≥10 in the tissue analysed, the corresponding MAF being 7 and 1.4%, in the minimum (70) and the maximum (361) sample size, respectively (16). In our simulations, we found that eQTLs with a small MAF identified in low sample sizes were highly likely to be false positives, regardless of which multiple testing correction strategy was used (Figure 2; Supplementary Figures S1A and S2). Based on above, when 100, 200 and 500 samples are available (typical in eQTL studies), we recommend a MAF cut-off at 10, 5 and 1%, respectively. Many studies listed in Supplementary Table S1 had a lower MAF cut-off than recommended. Detecting rare eQTLs with MAF 0.5% is possible in ≥2000 samples, but even 5000 samples cannot provide sufficient power unless the eQTL effect size is extremely high: ≥1 s.d. gene expression per allele dosage (Figures 2 and 3).

Recent eQTL studies have used pooled FDR methods to correct for multiple testing (46–50). Here, we show that pooled methods are inappropriate for eQTL studies, as they give inflated (sometimes substantially) FDR that worsen as sample size or eSNP MAF increases (Supplementary Figure S1). This suggests that many eQTLs identified in these studies may be false positives. Hierarchical multiple testing correction procedures had substantially better calibrated FDR. A hierarchical approach of permutation as local correction method followed by ST global adjustment is commonly used in eQTL studies (e.g. by GTEx (16)). When permutation was used as a local correction method, ST often had FDR slightly higher than the desired level in our simulations, while use of BH instead would have better calibrated FDR. Notably, ST and BH adjustment of multiple genes after correction for multiple local SNPs at each gene using other methods, except permutation tests, had identical results; therefore, we recommend using BH to adjust across genes rather than ST.

Most hierarchical procedures had nearly identical sensitivity when BH was used to correct for multiple testing across genes, thus FDR was a differentiating factor (Figure 2). Here, when studies were appropriately powered, eigenMT-BH was the most closely calibrated approach for controlling FDR at 5%, and it had the least variable FDR across different sample sizes. Although eigenMT-BH had FDR inflated above 5% in our simulations of proximal correlated genes and recessive causal eSNPs (Supplementary Figures S6A and S8A), these simulations represent worst case scenarios rather than realistic data. We expect only a fraction of eQTLs to comprise recessive effects, nor do we expect all causal eSNPs to regulate all genes, which are highly correlated, within a 1Mb window. Thus, we expect eigenMT-BH should control FDR at 5% in real eQTL datasets. On the other hand, Bonferroni-BH had the smallest FDR with negligibly lower sensitivity. The trade-offs between the use of Bonferroni-BH versus eigenMT-BH are best considered in the context of the specific study. Statistically, calibration is perhaps the deciding factor if the analysis is intended to guide time-consuming experimental follow-up of specific eQTLs, then it may be preferable to minimize FDR for a given detection power.

After eGene detection, identification of the causal eSNP among the significant eSNPs with high LD remains a challenge. Interestingly, we found that the most significant eSNP was the simulated causal eSNP ∼90% of the time. When the top variant was not the causal variant, ∼80% of the time the top eSNP was in high LD (*r*^2^ ≥ 0.8). The proportion of sentinel variants that were the causal eSNP was slightly lower, 80%, in conditional analyses applied in simulations of multiple causal eSNPs, motivating the use of fine-mapping approaches when there is evidence for multiple independent causal eSNPs.

Winner’s Curse in eQTL effect size estimation must be taken into account when comparing effect sizes from different tissue types or conditions, estimating replication sample size, or constructing predictive models. For example, a recent study compared *cis*-eQTL effects between blood samples (*N* = 1240 samples) and four other tissues (*N* < 85 samples), identifying >2000 probes with *cis-*eQTL associations that were tissue-dependent, and nearly half were with the same eSNP but with a different effect size (51). This may be an artefact of Winner’s Curse. To address eQTL effect over-estimation, we have presented a bootstrap method and tool for re-estimation, which should enable more accurate eQTL comparisons as well as predictive genetic models for gene expression for less accessible tissues, cell types, conditions or other situations where power is limited.

Since most eQTL studies focus on *cis*-eQTL mapping, there are limited findings of *trans*-eQTLs, thus realistic simulation of *trans*-eQTL datasets remains a challenge. Many of the multiple testing correction methods evaluated in our simulations are designed for *cis*-eQTL mapping only, such as those involving FastQTL and eigenMT. To deal with the multiple testing problem in *trans*-eQTL analysis, permutations would be time consuming for a whole genome scan, and one might consider estimating the number of independent gene expression traits and applying a Bonferroni correction. In terms of Winner’s Curse in effect size estimation, the bootstrap approach to reduce the upward bias would still be applicable in a *trans*-eQTL setting.

The investigation of the genetic component of transcriptional variation has become an essential part of linking genotype to phenotype (52). Despite the increasing scale of eQTL studies (e.g. 5257 samples in Yao *et al.* (6) and 2116 in Zhernakova *et al.* (14)), fundamental questions about study design and analysis strategies have remained unanswered. Here, we have investigated the sensitivity and FDR of diverse multiple testing strategies, the factors contributing the identification of the causal eSNP and the correction of eQTL effect size overestimation using a simple tool, BootstrapQTL. The insights from our simulation study are likely not limited to eQTL analysis and may extend to other studies of genome-related quantitative traits, such as chromatin accessibility, methylation and other epigenetic traits.

## Supplementary Material

Supplementary Data

## References

[1] Visscher P.M., Wray N.R., Zhang Q., Sklar P., McCarthy M.I., Brown M.A., Yang J. 10 years of GWAS Discovery: biology, function, and translation. Am. J. Hum. Genet. 2017; 101:5–22. 28686856 10.1016/j.ajhg.2017.06.005 PMC5501872

[2] Maurano M.T., Humbert R., Rynes E., Thurman R.E., Haugen E., Wang H., Reynolds A.P., Sandstrom R., Qu H., Brody J. Systematic localization of common disease-associated variation in regulatory DNA. Science. 2012; 337:1190–1195. 22955828 10.1126/science.1222794 PMC3771521

[3] Emilsson V., Thorleifsson G., Zhang B., Leonardson A.S., Zink F., Zhu J., Carlson S., Helgason A., Walters G.B., Gunnarsdottir S. Genetics of gene expression and its effect on disease. Nature. 2008; 452:423–428. 18344981 10.1038/nature06758

[4] Hoglinger G.U., Melhem N.M., Dickson D.W., Sleiman P.M.A., Wang L.-S., Klei L., Rademakers R., de Silva R., Litvan I., Riley D.E. Identification of common variants influencing risk of the tauopathy progressive supranuclear palsy. Nat. Genet. 2011; 43:699–705. 21685912 10.1038/ng.859 PMC3125476

[5] Franzen O., Ermel R., Cohain A., Akers N.K., Bi Narzo A., Talukdar H.A., Foroughi Asl H., Giambartolomei C., Fullard J.F., Sukhavasi K. Cardiometabolic risk loci share downstream cis- and trans-gene regulation across tissues and diseases. Science. 2016; 353:827–830. 27540175 10.1126/science.aad6970 PMC5534139

[6] Yao C., Joehanes R., Johnson A.D., Huan T., Liu C., Freedman J.E., Munson P.J., Hill D.E., Vidal M., Levy D. Dynamic role of trans regulation of gene expression in relation to complex traits. Am. J. Hum. Genet. 2017; 100:985–986. 28575653 10.1016/j.ajhg.2017.05.002 PMC5883609

[7] Nicolae D.L., Gamazon E., Zhang W., Duan S., Dolan M.E., Cox N.J. Trait-associated SNPs are more likely to be eQTLs: annotation to enhance discovery from GWAS. PLoS Genet. 2010; 6:e1000888. 20369019 10.1371/journal.pgen.1000888 PMC2848547

[8] Stranger B.E., Forrest M.S., Clark A.G., Minichiello M.J., Deutsch S., Lyle R., Hunt S., Kahl B., Antonarakis S.E., Tavare S. Genome-wide associations of gene expression variation in humans. PLoS Genet. 2005; 1:e78. 16362079 10.1371/journal.pgen.0010078 PMC1315281

[9] Flutre T., Wen X., Pritchard J., Stephens M. A statistical framework for joint eQTL analysis in multiple tissues. PLoS Genet. 2013; 9:e1003486. 23671422 10.1371/journal.pgen.1003486 PMC3649995

[10] Sun W. A statistical framework for eQTL mapping using RNA-seq data. Biometrics. 2012; 68:1–11. 21838806 10.1111/j.1541-0420.2011.01654.x PMC3218220

[11] Stegle O., Parts L., Durbin R., Winn J. A Bayesian framework to account for complex non-genetic factors in gene expression levels greatly increases power in eQTL studies. PLoS Comput. Biol. 2010; 6:e1000770. 20463871 10.1371/journal.pcbi.1000770 PMC2865505

[12] Fusi N., Stegle O., Lawrence N.D. Joint modelling of confounding factors and prominent genetic regulators provides increased accuracy in genetical genomics studies. PLoS Comput. Biol. 2012; 8:e1002330. 22241974 10.1371/journal.pcbi.1002330 PMC3252274

[13] Zhang L., Kim S. Learning gene networks under SNP perturbations using eQTL datasets. PLoS Comput. Biol. 2014; 10:e1003420. 24586125 10.1371/journal.pcbi.1003420 PMC3937098

[14] Zhernakova D.V., Deelen P., Vermaat M., van Iterson M., van Galen M., Arindrarto W., van ’t Hof P., Mei H., van Dijk F., Westra H.-J. Identification of context-dependent expression quantitative trait loci in whole blood. Nat. Genet. 2017; 49:139–145. 27918533 10.1038/ng.3737

[15] The GTEx Consortium Human genomics. The Genotype-Tissue Expression (GTEx) pilot analysis: multitissue gene regulation in humans. Science. 2015; 348:648–660. 25954001 10.1126/science.1262110 PMC4547484

[16] The GTEx Consortium Genetic effects on gene expression across human tissues. Nature. 2017; 550:204–213. 29022597 10.1038/nature24277 PMC5776756

[17] Benjamini Y., Hochberg Y. Controlling the false discovery rate: a practical and powerful approach to multiple testing. J. R. Stat. Soc. B. 1995; 57:289–300.

[18] Benjamini Y., Yekutieli D. The control of the false discovery rate in multiple testing under dependency. Ann. Stat. 2001; 29:1165–1188.

[19] Storey J.D., Tibshirani R. Statistical significance for genomewide studies. Proc. Natl. Acad. Sci. U.S.A. 2003; 100:9440–9445. 12883005 10.1073/pnas.1530509100 PMC170937

[20] Ongen H., Buil A., Brown A.A., Dermitzakis E.T., Delaneau O. Fast and efficient QTL mapper for thousands of molecular phenotypes. Bioinformatics. 2016; 32:1479–1485. 26708335 10.1093/bioinformatics/btv722 PMC4866519

[21] Sul J.H., Raj T., de Jong S., de Bakker P.I.W., Raychaudhuri S., Ophoff R.A., Stranger B.E., Eskin E., Han B. Accurate and fast multiple-testing correction in eQTL studies. Am. J. Hum. Genet. 2015; 96:857–868. 26027500 10.1016/j.ajhg.2015.04.012 PMC4457958

[22] Davis J.R., Fresard L., Knowles D.A., Pala M., Bustamante C.D., Battle A., Montgomery S.B. An efficient Multiple-Testing adjustment for eQTL studies that accounts for linkage disequilibrium between variants. Am. J. Hum. Genet. 2016; 98:216–224. 26749306 10.1016/j.ajhg.2015.11.021 PMC4716687

[23] Peterson C.B., Bogomolov M., Benjamini Y., Sabatti C. TreeQTL: hierarchical error control for eQTL findings. Bioinformatics. 2016; 32:2556–2558. 27153635 10.1093/bioinformatics/btw198 PMC4978936

[24] Gamazon E.R., Wheeler H.E., Shah K.P., Mozaffari S.V., Aquino-Michaels K., Carroll R.J., Eyler A.E., Denny J.C., Nicolae D.L., Cox N.J. A gene-based association method for mapping traits using reference transcriptome data. Nat. Genet. 2015; 47:1091–1098. 26258848 10.1038/ng.3367 PMC4552594

[25] Schadt E.E., Woo S., Hao K. Bayesian method to predict individual SNP genotypes from gene expression data. Nat. Genet. 2012; 44:603–608. 22484626 10.1038/ng.2248

[26] Garner C. Upward bias in odds ratio estimates from genome-wide association studies. Genet. Epidemiol. 2007; 31:288–295. 17266119 10.1002/gepi.20209

[27] Zöllner S., Pritchard J.K. Overcoming the Winner's Curse: estimating penetrance parameters from case-control data. Am. J. Hum. Genet. 2007; 80:605–615. 17357068 10.1086/512821 PMC1852705

[28] Ioannidis J.P.A., Thomas G., Daly M.J. Validating, augmenting and refining genome-wide association signals. Nat. Rev. Genet. 2009; 10:318–329. 19373277 10.1038/nrg2544 PMC7877552

[29] Forstmeier W., Schielzeth H. Cryptic multiple hypotheses testing in linear models: overestimated effect sizes and the Winner's Curse. Behav. Ecol. Sociobiol. 2011; 65:47–55. 21297852 10.1007/s00265-010-1038-5 PMC3015194

[30] Palmer C., Pe’er I. Statistical correction of the Winner's Curse explains replication variability in quantitative trait genome-wide association studies. PLoS Genet. 2017; 13:e1006916. 28715421 10.1371/journal.pgen.1006916 PMC5536394

[31] Spencer C.C.A., Su Z., Donnelly P., Marchini J. Designing genome-wide association studies: sample size, power, imputation, and the choice of genotyping chip. PLoS Genet. 2009; 5:e1000477. 19492015 10.1371/journal.pgen.1000477 PMC2688469

[32] Skol A.D., Scott L.J., Abecasis G.R., Boehnke M. Joint analysis is more efficient than replication-based analysis for two-stage genome-wide association studies. Nat. Genet. 2006; 38:209–213. 16415888 10.1038/ng1706

[33] Su Z., Marchini J., Donnelly P. HAPGEN2: simulation of multiple disease SNPs. Bioinformatics. 2011; 27:2304–2305. 21653516 10.1093/bioinformatics/btr341 PMC3150040

[34] Auton A., Brooks L.D., Durbin R.M., Garrison E.P., Kang H.M., Korbel J.O., Marchini J.L., McCarthy S., McVean G.A., Abecasis G.R. A global reference for human genetic variation. Nature. 2015; 526:68–74. 26432245 10.1038/nature15393 PMC4750478

[35] Inouye M., Silander K., Hamalainen E., Salomaa V., Harald K., Jousilahti P., Mannisto S., Eriksson J.G., Saarela J., Ripatti S. An immune response network associated with blood lipid levels. PLoS Genet. 2010; 6:e1001113. 20844574 10.1371/journal.pgen.1001113 PMC2936545

[36] Inouye M., Kettunen J., Soininen P., Silander K., Ripatti S., Kumpula L.S., Hamalainen E., Jousilahti P., Kangas A.J., Mannisto S. Metabonomic, transcriptomic, and genomic variation of a population cohort. Mol. Syst. Biol. 2010; 6:441. 21179014 10.1038/msb.2010.93 PMC3018170

[37] Aulchenko Y.S., Ripke S., Isaacs A., van Duijn C.M. GenABEL: an R library for genome-wide association analysis. Bioinformatics. 2007; 23:1294–1296. 17384015 10.1093/bioinformatics/btm108

[38] Jansen R., Hottenga J.-J., Nivard M.G., Abdellaoui A., Laport B., de Geus E.J., Wright F.A., Penninx B.W.J.H., Boomsma D.I. Conditional eQTL analysis reveals allelic heterogeneity of gene expression. Hum. Mol. Genet. 2017; 26:1444–1451. 28165122 10.1093/hmg/ddx043 PMC6075455

[39] Shabalin A.A. Matrix eQTL: ultra fast eQTL analysis via large matrix operations. Bioinformatics. 2012; 28:1353–1358. 22492648 10.1093/bioinformatics/bts163 PMC3348564

[40] R Core Team R: A language and environment for statistical computing. 2015; Vienna: R Foundation for Statistical Computing.

[41] Dabney A., Storey J.D. qvalue: Q-value estimation for false discovery rate control. R package version 2.8.0 https://bioconductor.org/packages/release/bioc/html/qvalue.html.

[42] Delaneau O., Ongen H., Brown A.A., Fort A., Panousis N.I., Dermitzakis E.T. A complete tool set for molecular QTL discovery and analysis. Nat. Commun. 2017; 8:15452. 28516912 10.1038/ncomms15452 PMC5454369

[43] Sun L., Bull S.B. Reduction of selection bias in genomewide studies by resampling. Genet. Epidemiol. 2005; 28:352–367. 15761913 10.1002/gepi.20068

[44] Peterson C.B., Bogomolov M., Benjamini Y., Sabatti C. Many phenotypes without many false Discoveries: Error controlling strategies for multitrait association studies. Genet. Epidemiol. 2016; 40:45–56. 26626037 10.1002/gepi.21942 PMC4738479

[45] Sun L., Dimitromanolakis A., Faye L.L., Paterson A.D., Waggott D., Bull S.B. BR-squared: a practical solution to the Winner's Curse in genome-wide scans. Hum. Genet. 2011; 129:545–552. 21246217 10.1007/s00439-011-0948-2 PMC3074069

[46] Sajuthi S.P., Sharma N.K., Chou J.W., Palmer N.D., McWilliams D.R., Beal J., Comeau M.E., Ma L., Calles-Escandon J., Demons J. Mapping adipose and muscle tissue expression quantitative trait loci in African Americans to identify genes for type 2 diabetes and obesity. Hum. Genet. 2016; 135:869–880. 27193597 10.1007/s00439-016-1680-8 PMC4947558

[47] Kirsten H., Al-Hasani H., Holdt L., Gross A., Beutner F., Krohn K., Horn K., Ahnert P., Burkhardt R., Reiche K. Dissecting the genetics of the human transcriptome identifies novel trait-related trans-eQTLs and corroborates the regulatory relevance of non-protein coding locidagger. Hum. Mol. Genet. 2015; 24:4746–4763. 26019233 10.1093/hmg/ddv194 PMC4512630

[48] Naranbhai V., Fairfax B.P., Makino S., Humburg P., Wong D., Ng E., Hill A.V.S., Knight J.C. Genomic modulators of gene expression in human neutrophils. Nat. Commun. 2015; 6:7545. 26151758 10.1038/ncomms8545 PMC4507005

[49] Ramasamy A., Trabzuni D., Guelfi S., Varghese V., Smith C., Walker R., De T., Coin L., de Silva R., Cookson M.R. Genetic variability in the regulation of gene expression in ten regions of the human brain. Nat. Neurosci. 2014; 17:1418–1428. 25174004 10.1038/nn.3801 PMC4208299

[50] Kim Y., Xia K., Tao R., Giusti-Rodriguez P., Vladimirov V., van den Oord E., Sullivan P.F. A meta-analysis of gene expression quantitative trait loci in brain. Transl. Psychiatry. 2014; 4:e459. 25290266 10.1038/tp.2014.96 PMC4350525

[51] Fu J., Wolfs M.G.M., Deelen P., Westra H.-J., Fehrmann R.S.N., te Meerman G.J., Buurman W.A., Rensen S.S.M., Groen H.J.M., Weersma R.K. Unraveling the regulatory mechanisms underlying tissue-dependent genetic variation of gene expression. PLoS Genet. 2012; 8:e1002431. 22275870 10.1371/journal.pgen.1002431 PMC3261927

[52] Albert F.W., Kruglyak L. The role of regulatory variation in complex traits and disease. Nat. Rev. Genet. 2015; 16:197–212. 25707927 10.1038/nrg3891

