# Supplementary material for: Power, false discovery rate and Winner’s Curse in eQTL studies
Source: Nucleic Acids Res. 2018 Sep 5;46(22):e133. doi: 10.1093/nar/gky780 (PMC6294523; doi:10.1093/nar/gky780)
Supplement: Supplementary Data [file gky780_supplemental_files.pdf]

Supplementary Tables and Figures

Supplementary Table 1: Sample size and minor allele frequency cut-off used in recent eQTL studies

Study	Tissue/cell type	Sample size	Minor allele frequency cut-off
Brynedal et al., 2017	lymphocyte cell lines	83~185	15%
GTEx consortium, 2017	various tissues (44)	70~361	1% and minor allele count ≥10
Kim-Hellmuth et al., 2017	monocytes (resting and stimulated)	134	5%
Ko et al., 2017	kidney	96	5%
Manry et al., 2017	whole blood (resting and stimulated)	51	10%
Ng et al., 2017	brain	494	1%
Pala et al., 2017	white blood cells	624	1%
Peng et al., 2017	placentas	159	minor allele count ≥5 (MAF ~1.6%)
Yao et al., 2017	whole blood	5257	1%
Chen et al., 2016	three immune cell types	197	minor allele count >4 (MAF ~1.2%)
Franzen et al., 2016	blood and six vascular and metabolic tissues	600	5%
Nedelec et al., 2016	macrophages (resting and stimulated)	172	5%
Quach et al., 2016	monocytes (resting and stimulated)	200	5%
Sajuthi et al., 2016	subcutaneous adipose and skeletal muscle	256 and 255	1%
Zhernakova et al., 2016	whole blood	2116	5%
Caliskan et al., 2015	peripheral blood mononuclear cells (resting and stimulated)	98	10%
Hulur et al., 2015	distal colonic samples	40	5%
Kirsten et al., 2015	peripheral blood mononuclear cells	2112	1%
Lock et al., 2015	blood and brain	43	5%
Naranbhai et al., 2015	peripheral blood CD16+ neutrophils	101	5%
Battle et al., 2014	whole blood	922	2.50%
Fairfax et al., 2014	monocytes (resting and stimulated)	432	4%
Hu et al., 2014	T cells (resting and stimulated)	174	1%
Kim et al., 2014	monocytes (resting and stimulated)	137	5%
Kim et al., 2014	brain	424	1%
Lee et al., 2014	dendritic cells (resting and stimulated)	534	5%
Ongen et al., 2014	tumour and normal colon mucosa samples	90	5%
Ramasamy et al., 2014	10 brain regions	134	5%
Wright et al., 2014	whole blood	2494	1%
Ye et al., 2014	CD4+ T cells (resting and stimulated)	348	5%
Garnier et al., 2013	monocytes	758	1%
Lappalainen et al., 2013	lymphocyte cell lines	462	5%
Li et al., 2013	tumour samples and normal blood samples	407	5%
Westra et al., 2013	non-transformed peripheral blood	5311	5%
Barreiro et al., 2012	dendritic cells (resting and stimulated)	65	10%
Fairfax et al., 2012	primary monotypes and primary B cells	283	1%

Fu et al., 2012	blood and four non-blood tissues	1240 and 62~77	5%
Grundberg et al., 2012	adipose, lymphocyte cell lines, and skin	667~777	5%
Hao et al., 2012	lung	1111	1%
Stranger et al., 2012	lymphocyte cell lines	726	5%

1. Brynedal, B. *et al.* Large-Scale trans-eQTLs Affect Hundreds of Transcripts and Mediate Patterns of Transcriptional Co-regulation. *Am J Hum Genet* **100,** 581–591 (2017).
2. GTEx Consortium. Genetic effects on gene expression across human tissues. *Nature* **550**, 204–213 (2017).
3. Kim-Hellmuth, S. & Lappalainen, T. Concerted Genetic Function in Blood Traits. *Cell* **167,** 1167–1169 (2016).
4. Ko, Y.-A. *et al.* Genetic-Variation-Driven Gene-Expression Changes Highlight Genes with Important Functions for Kidney Disease. *Am J Hum Genet* **100,** 940–953 (2017).
5. Manry, J. *et al.* Deciphering the genetic control of gene expression following Mycobacterium leprae antigen stimulation. *PLoS Genet* **13,** e1006952 (2017).
6. Ng, B. *et al.* An xQTL map integrates the genetic architecture of the human brain's transcriptome and epigenome. *Nat Neurosci* **20,** 1418–1426 (2017).
7. Pala, M. *et al.* Population- and individual-specific regulatory variation in Sardinia. *Nat Genet* **49,** 700–707 (2017).
8. Peng, S. *et al.* Expression quantitative trait loci (eQTLs) in human placentas suggest developmental origins of complex diseases. *Hum Mol Genet* **26,** 3432–3441 (2017).
9. Yao, C. *et al.* Dynamic Role of trans Regulation of Gene Expression in Relation to Complex Traits. *Am J Hum Genet* **100,** 571–580 (2017).
10. Chen, L. *et al.* Genetic Drivers of Epigenetic and Transcriptional Variation in Human Immune Cells. *Cell* **167,** 1398–1414.e24 (2016).
11. Franzen, O. *et al.* Cardiometabolic risk loci share downstream cis- and trans-gene regulation across tissues and diseases. *Science* **353,** 827–830 (2016).
12. Nedelec, Y. *et al.* Genetic Ancestry and Natural Selection Drive Population Differences in Immune Responses to Pathogens. *Cell* **167,** 657–669.e21 (2016).
13. Quach, H. *et al.* Genetic Adaptation and Neandertal Admixture Shaped the Immune System of Human Populations. *Cell* **167,** 643–656.e17 (2016).
14. Sajuthi, S. P. *et al.* Mapping adipose and muscle tissue expression quantitative trait loci in African Americans to identify genes for type 2 diabetes and obesity. *Hum Genet* **135,** 869–880 (2016).
15. Zhernakova, D. V. *et al.* Identification of context-dependent expression quantitative trait loci in whole blood. *Nat Genet* **49,** 139–145 (2017).
16. Caliskan, M., Baker, S. W., Gilad, Y. & Ober, C. Host genetic variation influences gene expression response to rhinovirus infection. *PLoS Genet* **11,** e1005111 (2015).
17. Hulur, I. *et al.* Enrichment of inflammatory bowel disease and colorectal cancer risk variants in colon expression quantitative trait loci. *BMC Genomics* **16,** 138 (2015).
18. Kirsten, H. *et al.* Dissecting the genetics of the human transcriptome identifies novel trait-related trans-eQTLs and corroborates the regulatory relevance of non-protein coding locidagger. *Hum Mol Genet* **24,** 4746–4763 (2015).
19. Lock, E. F. *et al.* Joint eQTL assessment of whole blood and dura mater tissue from individuals with Chiari type I malformation. *BMC Genomics* **16,** 11 (2015).
20. Naranbhai, V. *et al.* Genomic modulators of gene expression in human neutrophils. *Nat Commun* **6,** 7545 (2015).
21. Battle, A. *et al.* Characterizing the genetic basis of transcriptome diversity through RNA-sequencing of 922 individuals. *Genome Res* **24,** 14–24 (2014).
22. Fairfax, B. P. *et al.* Innate immune activity conditions the effect of regulatory variants upon monocyte gene expression. *Science* **343,** 1246949 (2014).
23. Hu, X. *et al.* Regulation of gene expression in autoimmune disease loci and the genetic basis of proliferation in CD4+ effector memory T cells. *PLoS Genet* **10,** e1004404 (2014).
24. Kim, S. *et al.* Characterizing the genetic basis of innate immune response in TLR4-activated human monocytes. *Nat Commun* **5,** 5236 (2014).
25. Kim, Y. *et al.* A meta-analysis of gene expression quantitative trait loci in brain. *Transl Psychiatry* **4,** e459 (2014).
26. Lee, M. N. *et al.* Common genetic variants modulate pathogen-sensing responses in human dendritic cells. *Science* **343,** 1246980 (2014).
27. Ongen, H. *et al.* Putative cis-regulatory drivers in colorectal cancer. *Nature* **512,** 87–90 (2014).
28. Ramasamy, A. *et al.* Genetic variability in the regulation of gene expression in ten regions of the human brain. *Nat Neurosci* **17,** 1418–1428 (2014).

29. Wright, F. A. *et al.* Heritability and genomics of gene expression in peripheral blood. *Nat Genet* **46,** 430–437 (2014).
30. Ye, C. J. *et al.* Intersection of population variation and autoimmunity genetics in human T cell activation. *Science* **345,** 1254665 (2014).
31. Garnier, S. *et al.* Genome-wide haplotype analysis of cis expression quantitative trait loci in monocytes. *PLoS Genet* **9,** e1003240 (2013).
32. Lappalainen, T. *et al.* Transcriptome and genome sequencing uncovers functional variation in humans. *Nature* **501,** 506–511 (2013).
33. Li, Q. *et al.* Integrative eQTL-based analyses reveal the biology of breast cancer risk loci. *Cell* **152,** 633–641 (2013).
34. Westra, H.-J. *et al.* Systematic identification of trans eQTLs as putative drivers of known disease associations. *Nat Genet* **45,** 1238–1243 (2013).
35. Barreiro, L. B. *et al.* Deciphering the genetic architecture of variation in the immune response to Mycobacterium tuberculosis infection. *Proc Natl Acad Sci U S A* **109,** 1204–1209 (2012).
36. Fairfax, B. P. *et al.* Genetics of gene expression in primary immune cells identifies cell type-specific master regulators and roles of HLA alleles. *Nat Genet* **44,** 502–510 (2012).
37. Fu, J. *et al.* Unraveling the regulatory mechanisms underlying tissue-dependent genetic variation of gene expression. *PLoS Genet* **8,** e1002431 (2012).
38. Grundberg, E. *et al.* Mapping cis- and trans-regulatory effects across multiple tissues in twins. *Nat Genet* **44,** 1084–1089 (2012).
39. Hao, K. *et al.* Lung eQTLs to help reveal the molecular underpinnings of asthma. *PLoS Genet* **8,** e1003029 (2012).
40. Stranger, B. E. *et al.* Patterns of cis regulatory variation in diverse human populations. *PLoS Genet* **8,** e1002639 (2012).

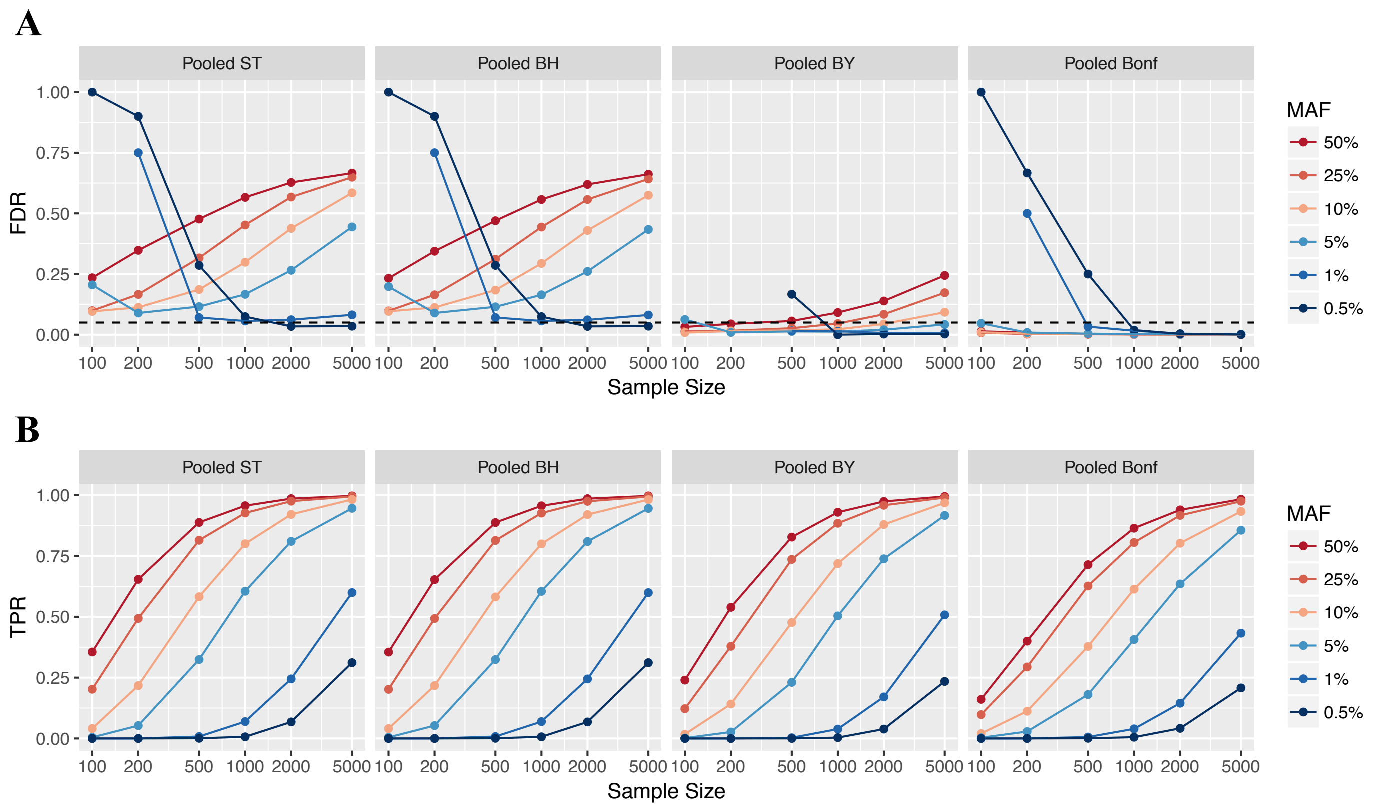

Figure S1. False discovery rate (FDR) and sensitivity of pooled methods for increasing sample sizes. Each panel represents a pooled multiple testing correction method, namely the Storey-Tibshirani (ST), Benjamini-Hochberg (BH), Benjamini-Yekutieli (BY) FDR procedures, and the Bonferroni correction procedure. FDR (**A**) and sensitivity/true positive rate (TPR) (**B**) are shown on y-axes and sample sizes are shown on x-axes. Each dot represents one simulation scenario and are coloured according to the minor allele frequency (MAF) of the simulated causal eSNPs. The dashed horizontal lines in panel A indicate the desired FDR level of 5%. Missing dots in panel A are scenarios in which no significant eGenes were identified (the corresponding sensitivity in panel B is 0).

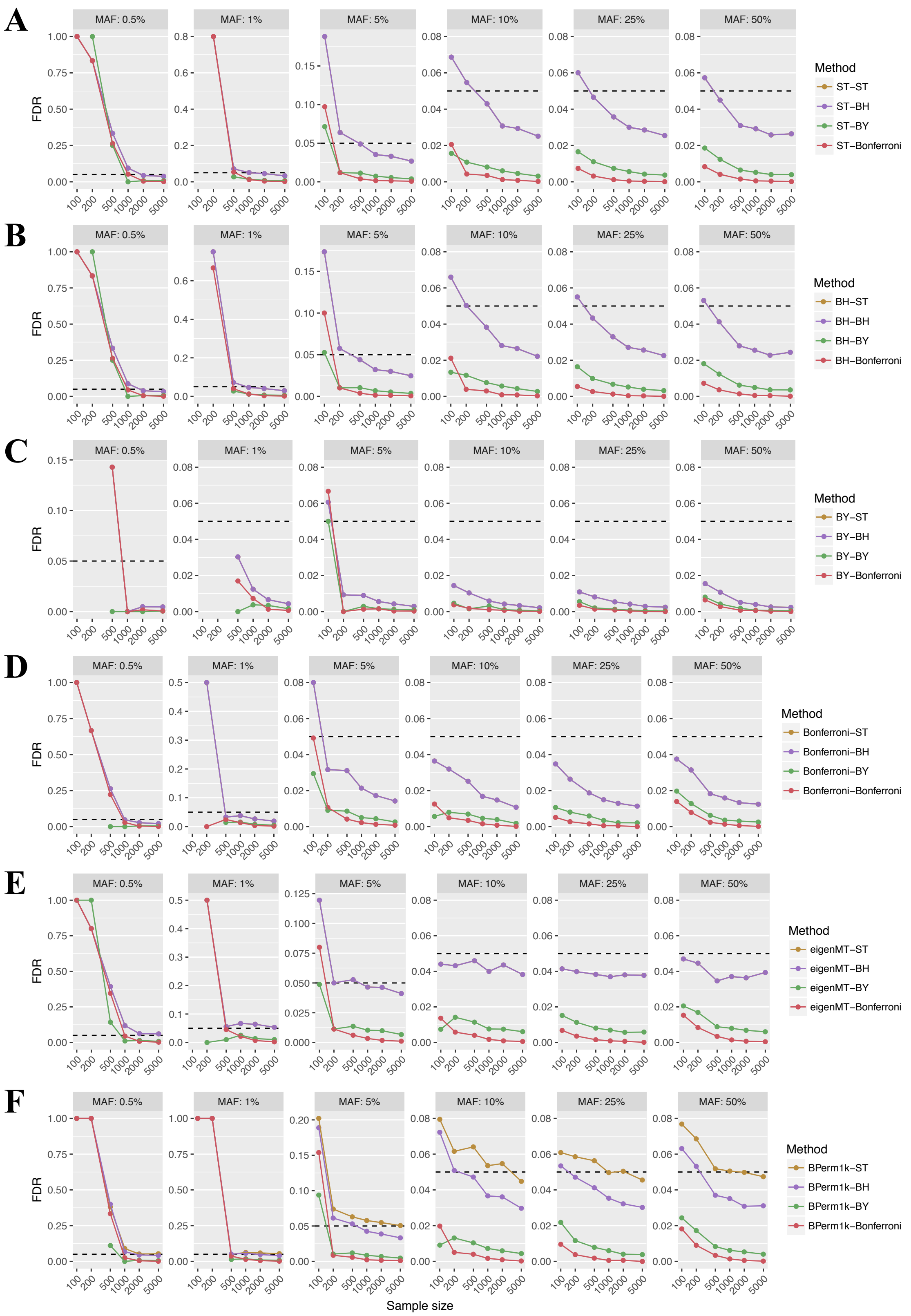

Figure S2. False discovery rate (FDR) of all hierarchical multiple testing correction methods. Each row compares four methods (different colours) applied in the global correction step of hierarchical correction procedures, which aims to correct for multiple genes being tested: the Storey-Tibshirani (ST), Benjamini-Hochberg (BH), Benjamini-Yekutieli (BY) FDR procedures, and the Bonferroni correction procedure. Rows represent different methods (ST, BH, BY, Bonferroni, eigenMT, and beta approximation based permutation approach) used in the local correction step of hierarchical correction procedures, which aims to correct for multiple SNPs tested for each gene. Scenarios where true eSNPs had different minor allele frequencies (MAFs) are shown in different plots and sample size is shown on x-axes. The dashed horizontal lines indicate the desired FDR level of 5%. Note that the use of ST and BH for global correction had the same FDR except when permutation approaches were used for local correction.

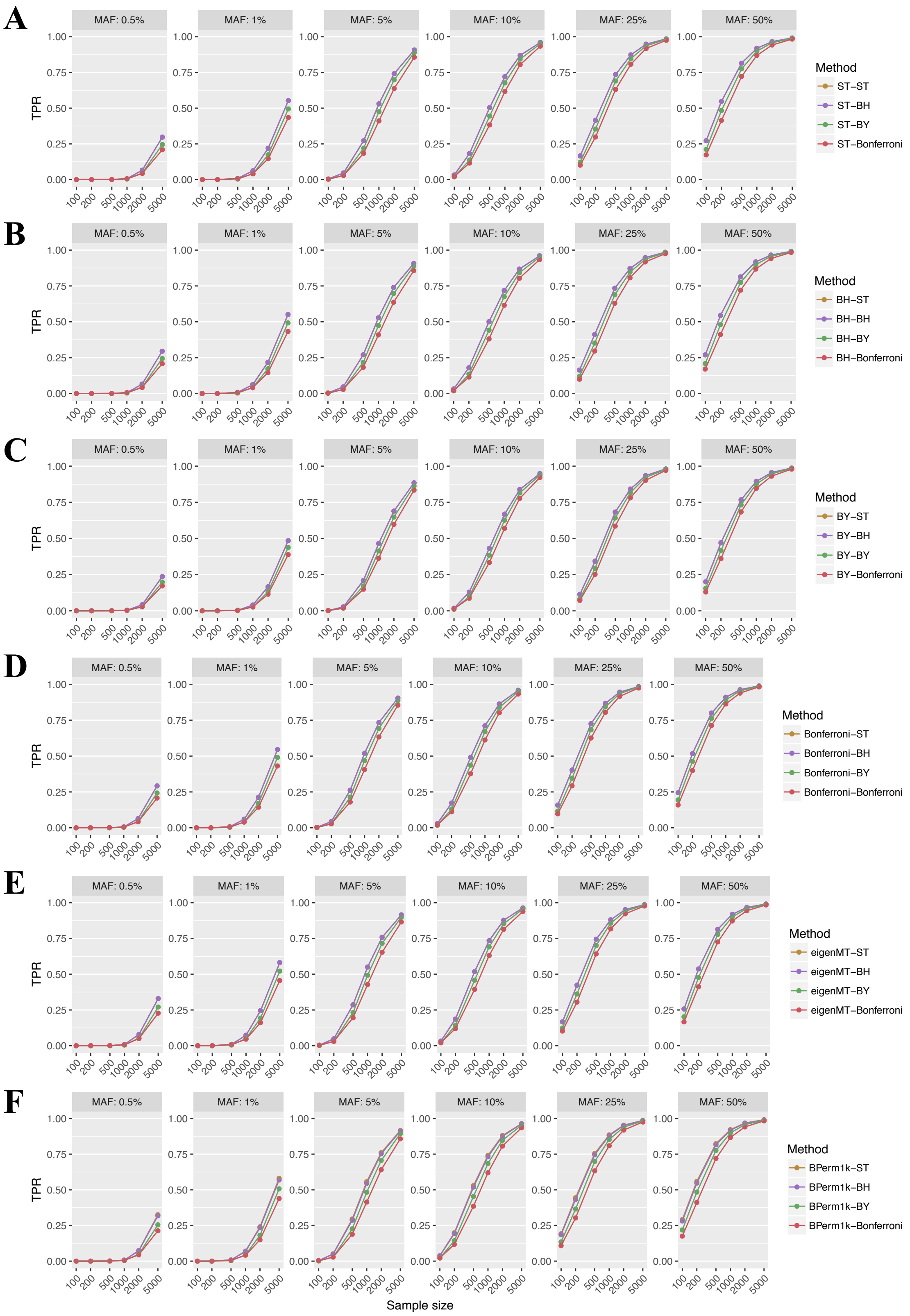

Figure S3. Sensitivity/true positive rate (TPR) of all hierarchical multiple testing correction methods. Each row compares four methods (different colours) applied in the global correction step of hierarchical correction procedures, which aims to correct for multiple genes being tested: the Storey-Tibshirani (ST), Benjamini-Hochberg (BH), Benjamini-Yekutieli (BY) false discovery rate (FDR) procedures, and the Bonferroni correction procedure. Rows represent different methods (ST, BH, BY, Bonferroni, eigenMT, and permutation approach) used in the local correction step of hierarchical correction procedures, which aims to correct for multiple SNPs tested for each gene. Scenarios where true eSNPs had different minor allele frequencies (MAFs) are shown in different plots and sample size is shown on x-axes. Note that use of ST and BH for global correction had the same sensitivity except when permutation approaches were used for local correction.

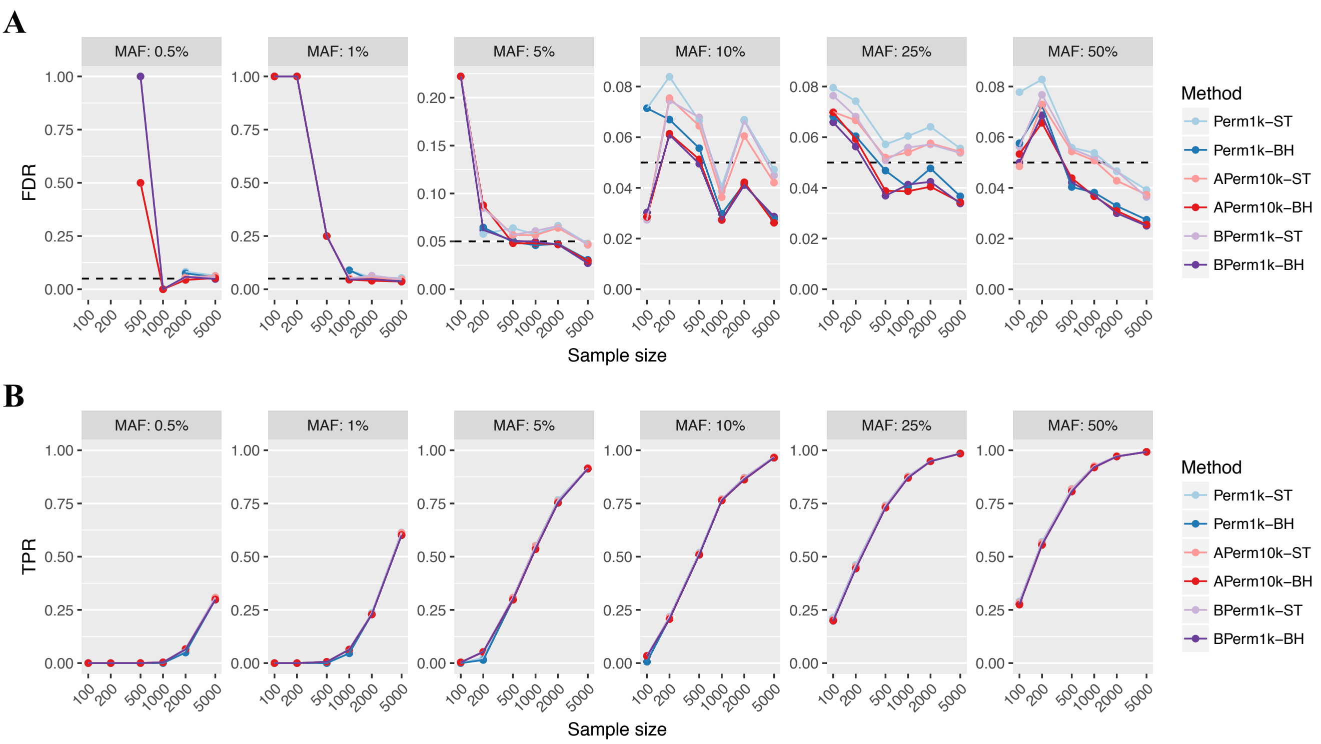

Figure S4. False discovery rate (FDR) and sensitivity of hierarchical multiple testing correction using permutation tests. FDR (**A**) and sensitivity/true positive rate (TPR) (**B**) of three permutation approaches (different colours) used in the local correction step of hierarchical correction procedures calculated from 10 simulations are shown on y-axes: using exact permutation p-values obtained from 1,000 permutations (Perm1k), p-values obtained from beta approximation from 1,000 permutations (BPerm1k), and p-values obtained from a beta approximation scheme with a minimum of 100 and maximum of 10,000 permutations (APerm10k). Benjamini-Hochberg (BH) or Storey-Tibshirani (ST) FDR procedure were used in the global correction step of hierarchical correction procedures to correct for multiple genes. Each dot represents one scenario and plots show scenarios with different causal eSNP MAFs. The horizontal lines in panel A indicate the desired FDR level of 5%. FDR was not calculated for scenarios where no significant eGenes were identified.

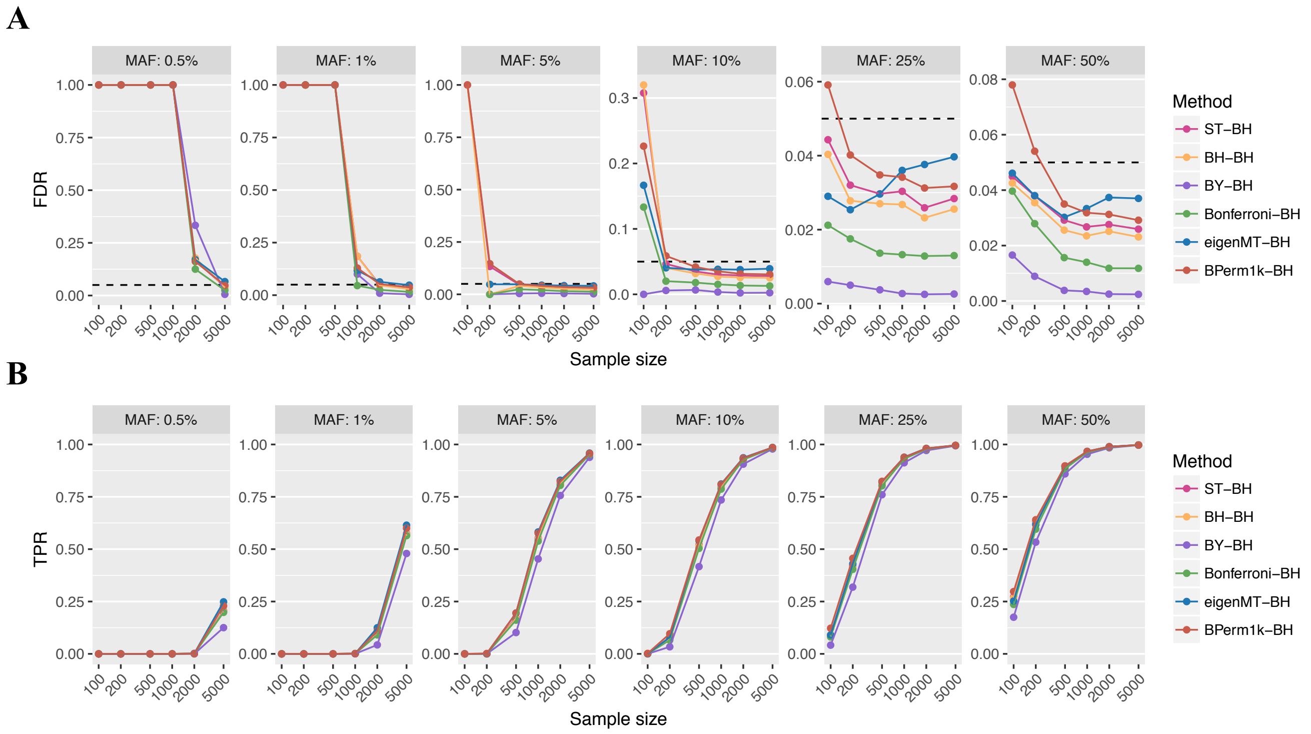

Figure S5. False discovery rate (FDR) and sensitivity of selected hierarchical multiple testing correction methods in simulations of log-normal noise. The gene expression data were inverse normal transformed. Comparison of the FDR (**A**) and sensitivity/true positive rate (TPR) (**B**) of six methods (different colours) for controlling multiple testing of SNPs at each gene (local correction), with Benjamini-Hochberg (BH) used to control for multiple testing across all genes (global correction). The six methods compared were Storey-Tibshirani (ST), Benjamini-Hochberg (BH), Benjamini-Yekutieli (BY), Bonferroni correction, eigenMT, and permutation tests based on beta approximation (BPerm1k). Each dot represents one scenario and plots show different minor allele frequencies (MAFs) of the simulated causal eSNPs. The dashed horizontal lines in panel A indicate the desired FDR level of 5%. Scenarios where no significant eGenes were identified are not shown in panel A.

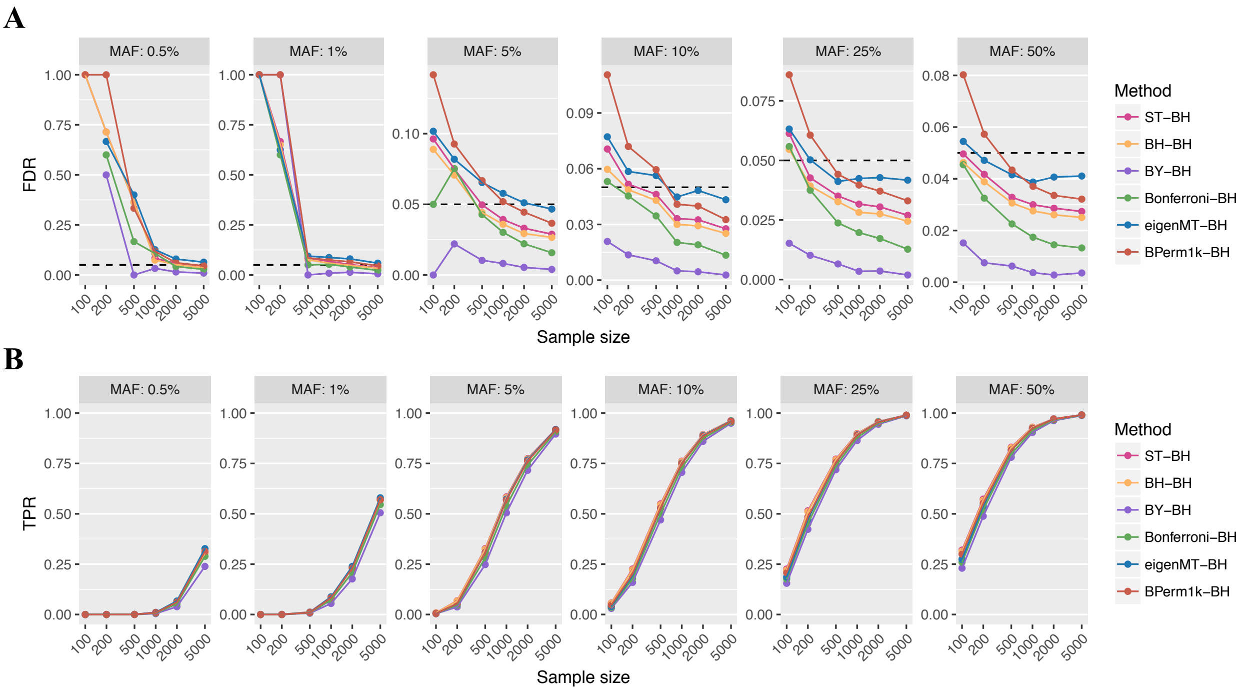

Figure S6. False discovery rate (FDR) and sensitivity of selected hierarchical multiple testing correction methods in simulations of correlated gene expression. Comparison of the FDR (**A**) and sensitivity/true positive rate (TPR) (**B**) of six methods (different colours) for controlling multiple testing of SNPs at each gene (local correction), with Benjamini-Hochberg (BH) used to control for multiple testing across all genes (global correction). The six methods compared were Storey-Tibshirani (ST), Benjamini-Hochberg (BH), Benjamini-Yekutieli (BY), Bonferroni correction, eigenMT, and permutation tests based on beta approximation (BPerm1k). Each dot represents one scenario and plots show different minor allele frequencies (MAFs) of the simulated causal eSNPs. The dashed horizontal lines in panel A indicate the desired FDR level of 5%. Scenarios where no significant eGenes were identified are not shown in panel A.

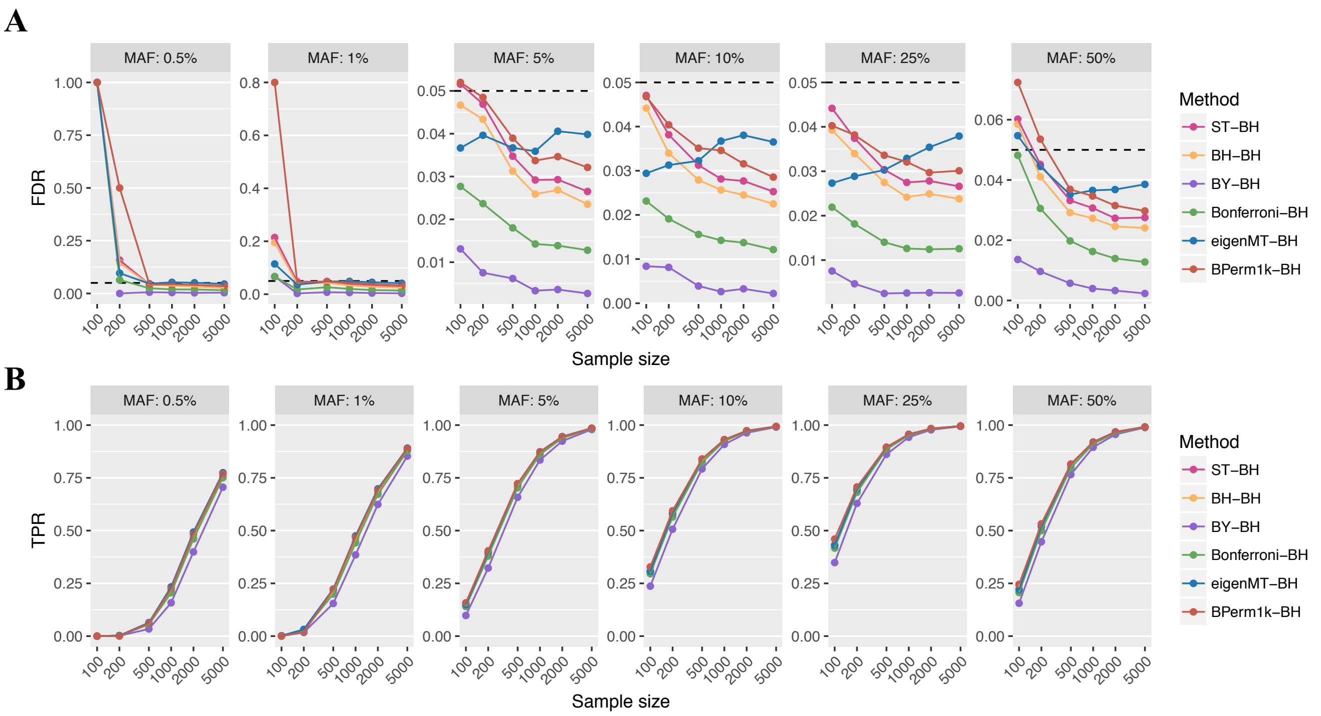

Figure S7. False discovery rate (FDR) and sensitivity of selected hierarchical multiple testing correction methods in simulations of dominant effects. Comparison of the FDR (**A**) and sensitivity/true positive rate (TPR) (**B**) of six methods (different colours) for controlling multiple testing of SNPs at each gene (local correction), with Benjamini-Hochberg (BH) used to control for multiple testing across all genes (global correction). The six methods compared were Storey-Tibshirani (ST), Benjamini-Hochberg (BH), Benjamini-Yekutieli (BY), Bonferroni correction, eigenMT, and permutation tests based on beta approximation (BPerm1k). Each dot represents one scenario and plots show different minor allele frequencies (MAFs) of the simulated causal eSNPs. The dashed horizontal lines in panel A indicate the desired FDR level of 5%. Scenarios where no significant eGenes were identified are not shown in panel A.

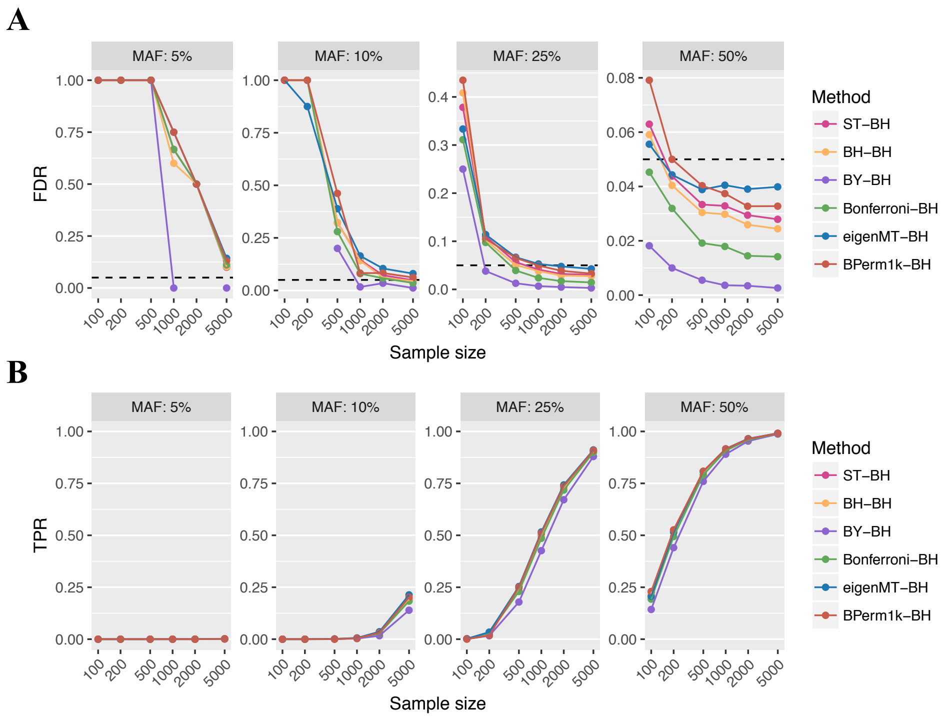

Figure S8. False discovery rate (FDR) and sensitivity of selected hierarchical multiple testing correction methods in simulations of recessive effects. Comparison of the FDR (**A**) and sensitivity/true positive rate (TPR) (**B**) of six methods (different colours) for controlling multiple testing of SNPs at each gene (local correction), with Benjamini-Hochberg (BH) used to control for multiple testing across all genes (global correction). The six methods compared were Storey-Tibshirani (ST), Benjamini-Hochberg (BH), Benjamini-Yekutieli (BY), Bonferroni correction, eigenMT, and permutation tests based on beta approximation (BPerm1k). Each dot represents one scenario and plots show different minor allele frequencies (MAFs) of the simulated causal eSNPs. The dashed horizontal lines in panel A indicate the desired FDR level of 5%. Scenarios where no significant eGenes were identified are not shown in panel A.

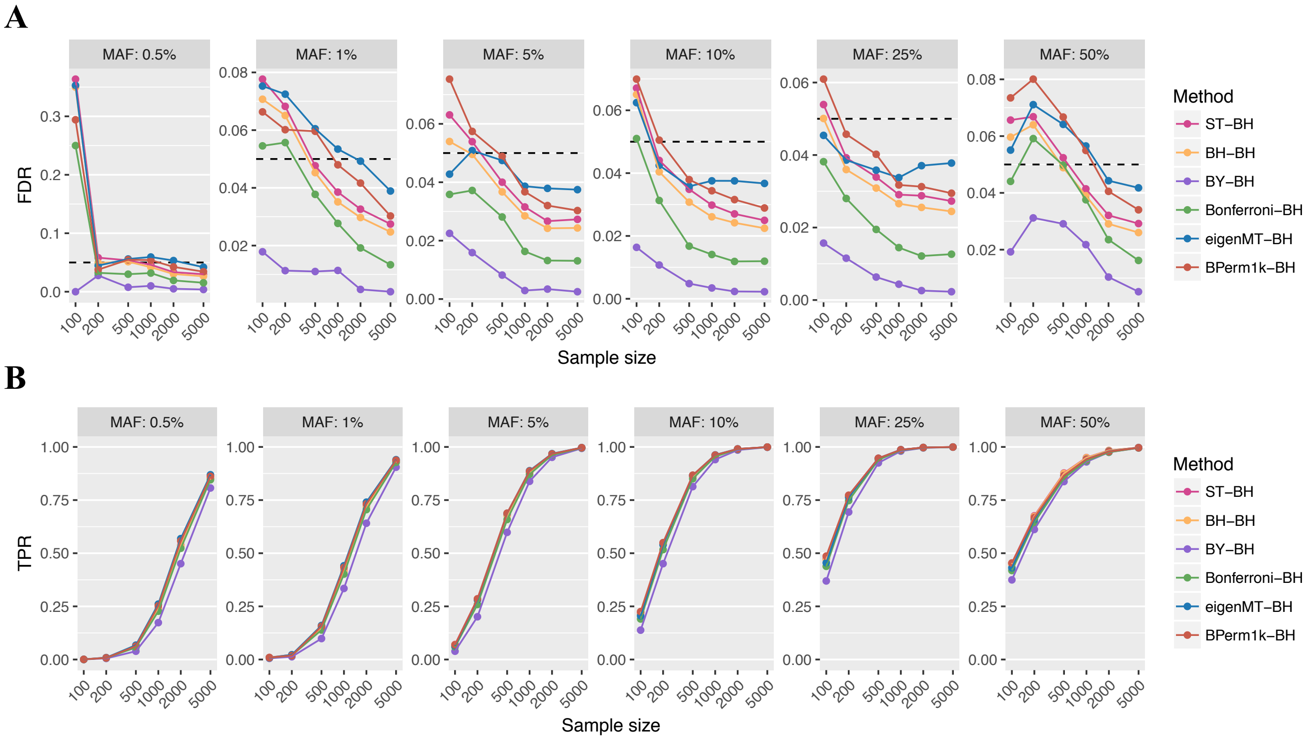

Figure S9. False discovery rate (FDR) and sensitivity of selected hierarchical multiple testing correction methods in simulations of two causal eSNPs per eGene. Comparison of the FDR (**A**) and sensitivity/true positive rate (TPR) (**B**) of six methods (different colours) for controlling multiple testing of SNPs at each gene (local correction), with Benjamini-Hochberg (BH) used to control for multiple testing across all genes (global correction). The six methods compared were Storey-Tibshirani (ST), Benjamini-Hochberg (BH), Benjamini-Yekutieli (BY), Bonferroni correction, eigenMT, and permutation tests based on beta approximation (BPerm1k). Each dot represents one scenario and scenarios where most simulated causal eSNPs had different minor allele frequencies (MAFs) are shown in different plots. The dashed horizontal lines in panel A indicate the desired FDR level of 5%. Scenarios where no significant eGenes were identified are not shown in panel A. We also simulated scenarios where each eGene had three causal eSNPs, and the results were the same.

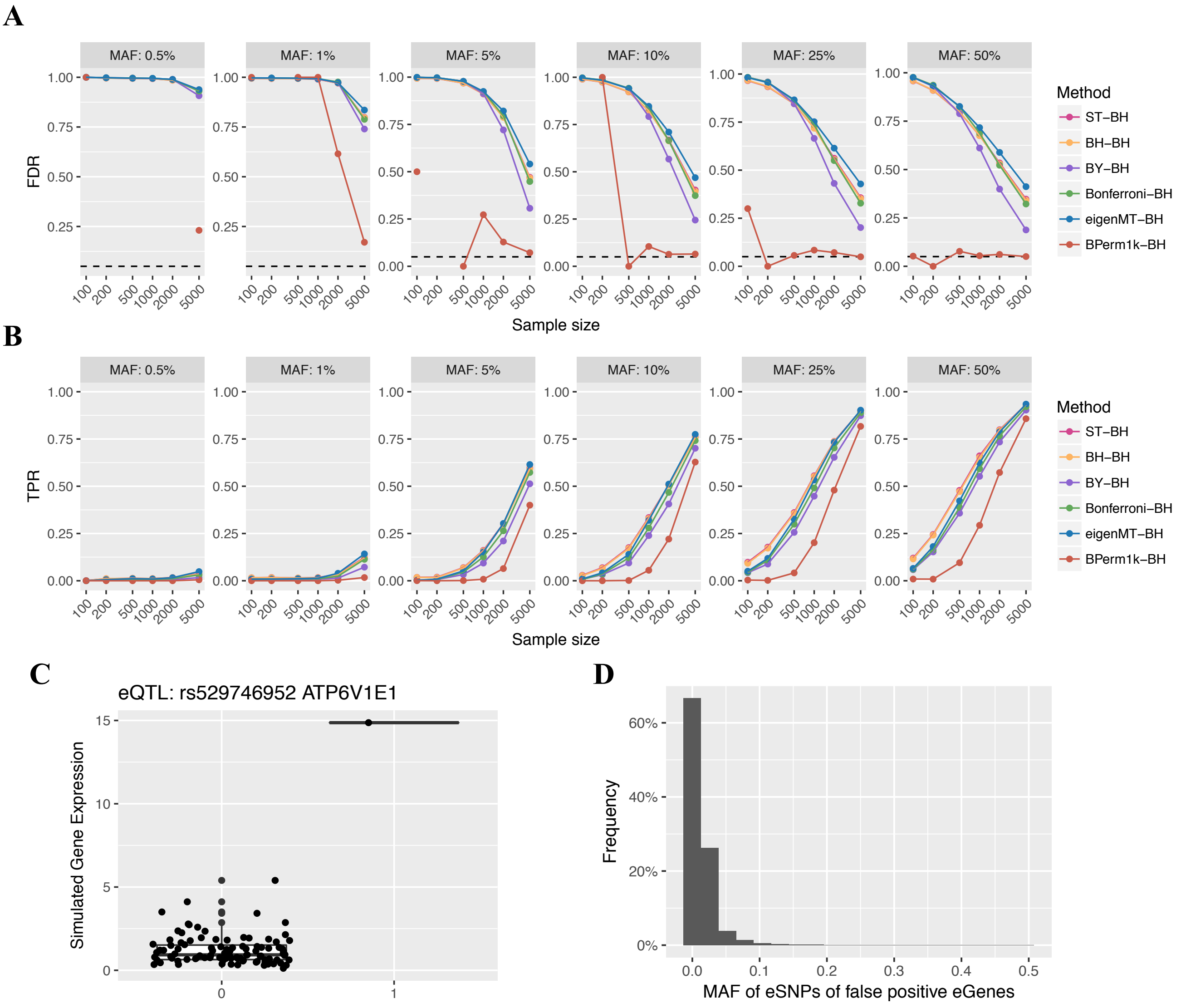

Figure S10. Simulations of log-normal noise without inverse normal transformation. Panel **A** and **B** show the false discovery rate (FDR) (**A**) and sensitivity/true positive rate (TPR) (**B**) of six methods (different colours) for controlling multiple testing of SNPs at each gene (local correction), with Benjamini-Hochberg (BH) used to control for multiple testing across all genes (global correction). The six methods compared were Storey-Tibshirani (ST), Benjamini-Hochberg (BH), Benjamini-Yekutieli (BY), Bonferroni correction, eigenMT, and permutation tests based on beta approximation (BPerm1k). Each dot represents one scenario and plots show different minor allele frequencies (MAFs) of the simulated causal eSNPs. The dashed horizontal lines in panel **A** indicate the desired FDR level of 5%. Scenarios where no significant eGenes were identified are not shown in panel **A**. Here the FDR and sensitivity were the average across ten replicates. Panel **C** shows an example of a typical false positive eQTL association, which was the most significant eGene and its most significant SNP in a scenario with a sample size of 100. Gene expression levels are shown on the y-axis. Each dot on the boxplot represents one individual, stratified by minor allele dosages on the x-axis. The eSNP shown here had a low frequency (0.5%) so there was no individual with homozygous for the minor allele. Most of the false positive eQTLs had low frequencies and the histogram in panel **D** shows the MAF distribution of significant eSNPs for false positive eGenes.

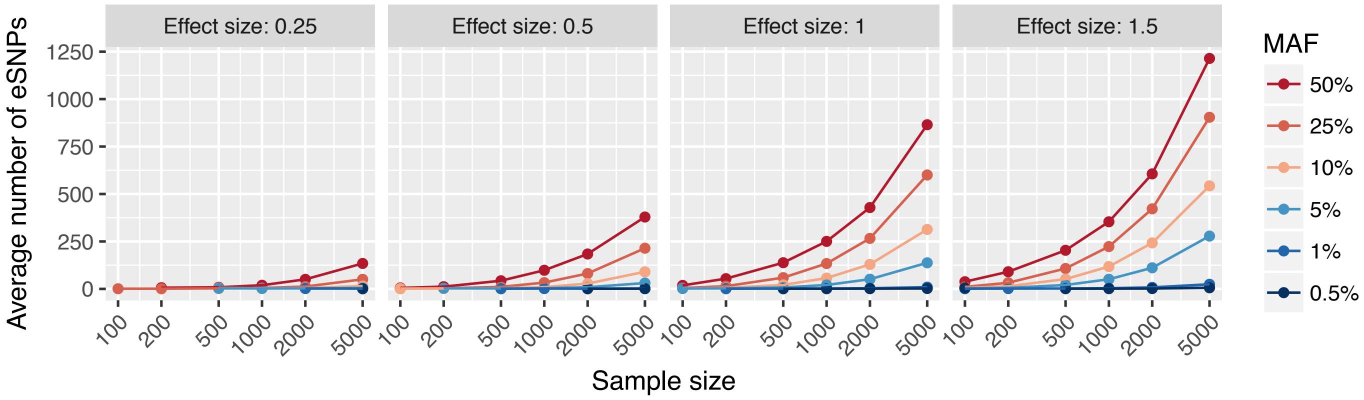

Figure S11. Average number of significant eSNPs per true positive eGene. For each scenario where a constant effect size (0.25, 0.5, 1.0, or 1.5 s.d. gene expression per allele) was simulated, the average number of eSNPs significantly associated with each true positive eGene was calculated (y-axis). A hierarchical correction procedure using eigenMT for local correction and BH for global correction (eigenMT-BH) was used to correct for multiple testing. Sample size is shown on x-axes and colors of dots indicate varying minor allele frequencies (MAFs) of true eSNPs.

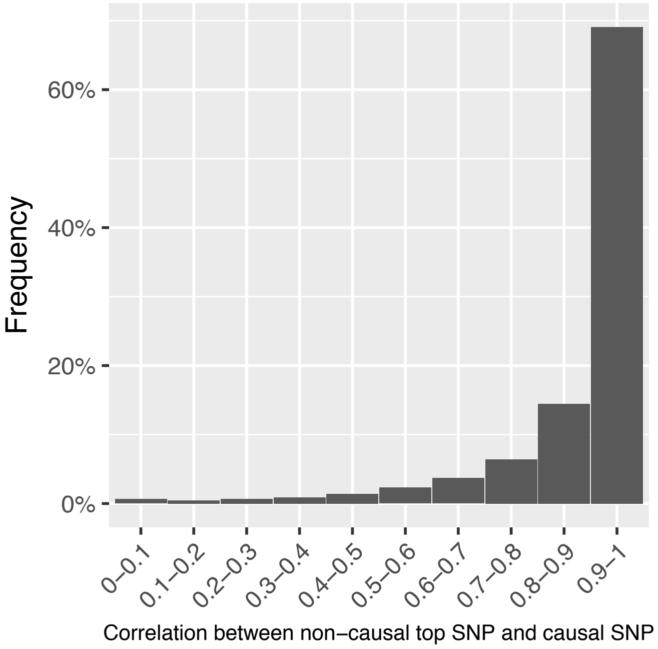

Figure S12. Histogram of linkage disequilibrium (LD) r^2 between the top SNPs that were not causal and their respective causal eSNPs. For every true positive eGene, we calculated the LD r^2 between the most significant SNP and the simulated causal eSNP. Histogram bins show the percentage of non-causal top SNPs with the given r^2 LD range to the causal eSNP calculated across 100 simulations of all 144 scenarios where a constant effect size was simulated. A hierarchical correction procedure using eigenMT for local correction and BH for global correction (eigenMT-BH) was used to correct for multiple testing. 83% of non-causal top SNPs had an r^2 ≥0.8 with their respective causal eSNP.

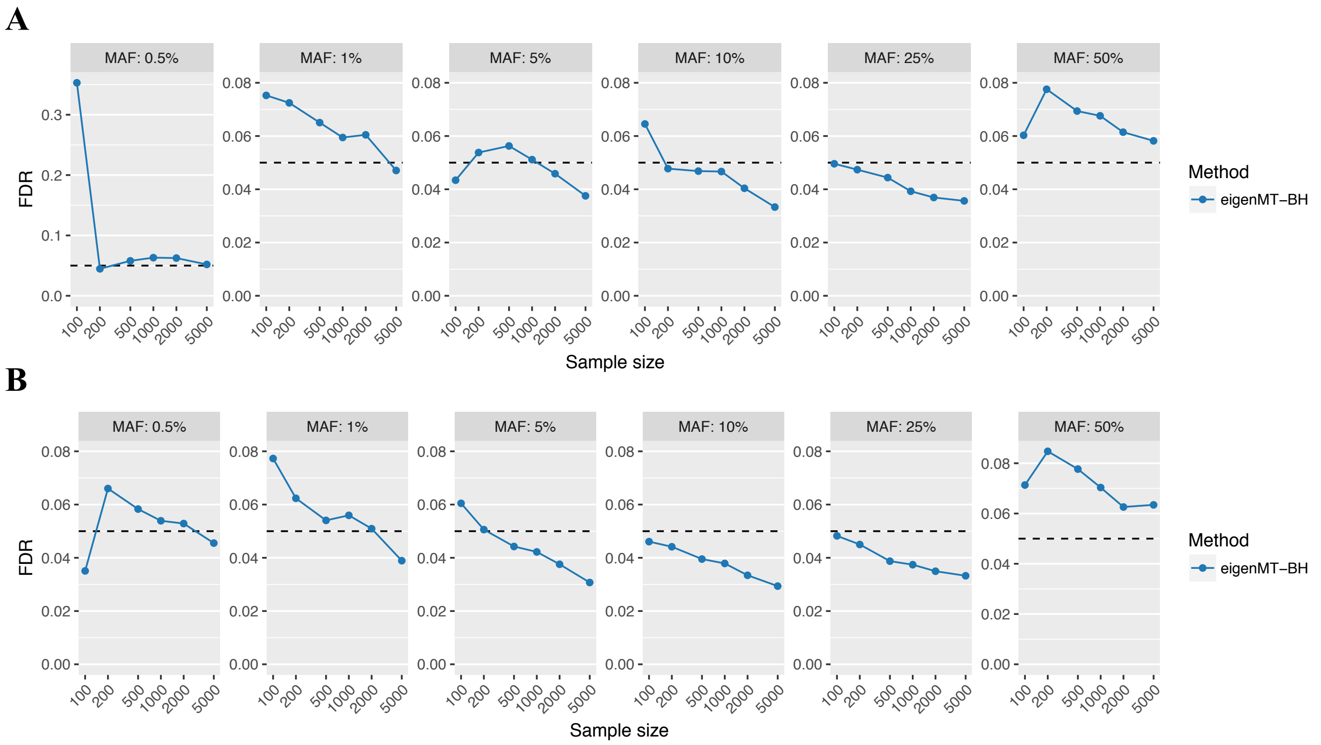

Figure S13. False discovery rate (FDR) among the independent eQTL signals identified by conditional analyses in simulations of two (A) or three (B) causal eSNPs per eGene. Each dot shows the rate of false positive eQTL signals among all independent eQTL signals identified in conditional analyses in one scenario. Sample sizes are shown on the x-axes and plots show different minor allele frequencies (MAFs) of the simulated causal eSNPs. The horizontal lines indicate the desired FDR level of 5%. The nominal significance thresholds in each scenario were determined by eigenMT-BH hierarchical correction procedure in the initial eQTL scan. An independent eQTL signal was taken as a FP if no significant SNPs were in high LD ($r^2 \geq 0.8$) with any simulated causal eSNP, or where an eQTL signal was not truly independent from another identified true eQTL signal (**Methods**).

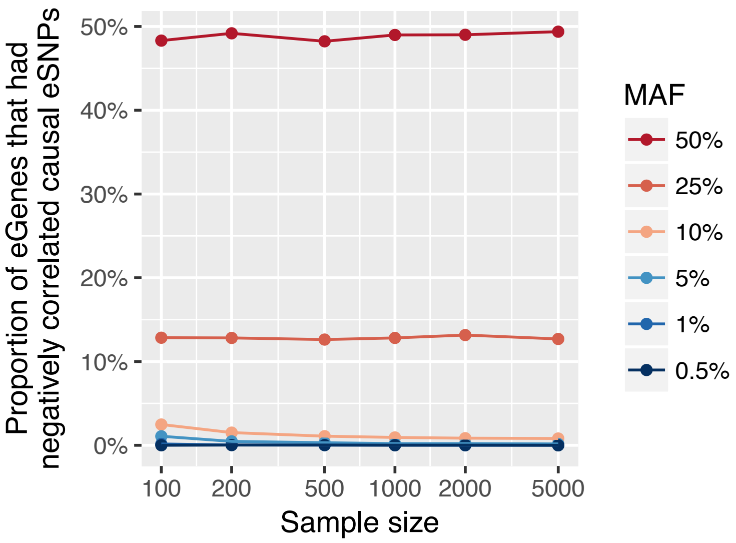

Figure S14. Proportion of eGenes that had simulated causal eSNPs with negatively correlated minor allele dosages in simulations of two causal eSNPs per eGene. Each dot represents one of the 36 scenarios and they are coloured based on minor allele frequencies (MAFs) of the causal eSNPs. Sample size is shown on the x-axis. For each eGene, the Pearson correlation between the two minor allele dosages of the causal eSNPs was calculated and the proportion of eGenes that had two causal eSNPs with negatively correlated minor allele dosages is shown on the y-axis. Here we only show the simulations where each eGene had two causal eSNPs; in simulations where each eGene had three causal eSNPs, the results were the same.

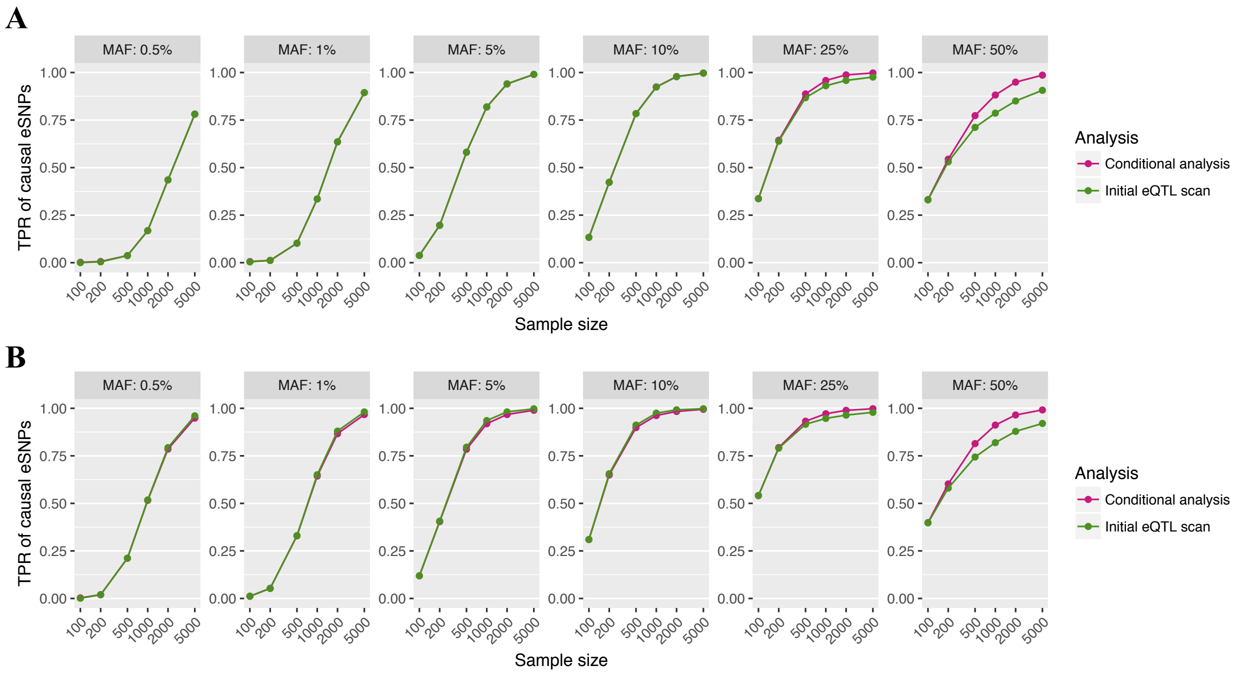

Figure S15. Proportion of simulated causal eSNPs identified in conditional analyses and the initial eQTL mapping step in simulations of two (A) or three (B) causal eSNPs per eGene. Each dot represents one scenario ordered by sample size (x-axes) and plots show different minor allele frequencies (MAFs) of simulated causal eSNPs. The rate of true positives (TPR, or sensitivity) identified among all 400 or 600 simulated causal eSNPs is shown on the y-axes. Results based on conditional analyses and the initial eQTL mapping are in pink and green, respectively. The nominal significance thresholds used in conditional analyses were determined by eigenMT-BH hierarchical correction procedure in the initial eQTL scan. A simulated causal eSNP was successfully identified (or treated as a true positive) if this causal eSNP or any SNPs in high LD ($r^2 \geq 0.8$) with it were significant.

A

B

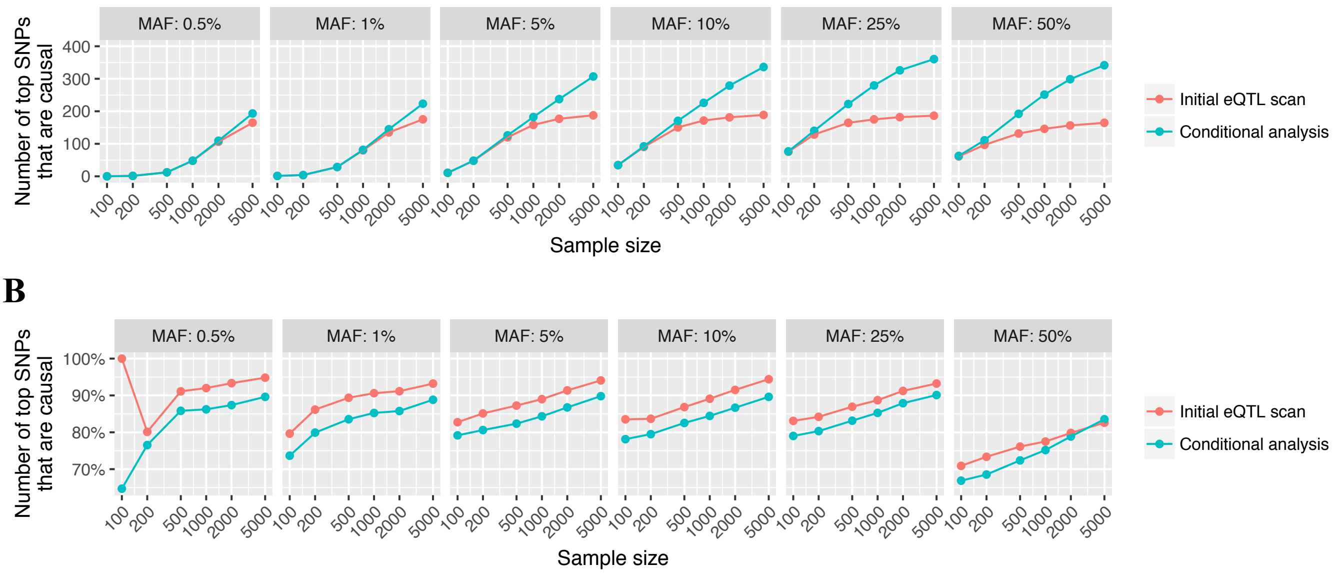

Figure S16. Identification of causal eSNPs among top SNPs in simulations of two causal eSNPs per eGene. Each dot represents one scenario in the order of increasing sample sizes (x-axes) and the plots show different minor allele frequencies (MAFs) of the simulated causal eSNPs. In panel **A**, the y-axes show the number of top SNPs that were the simulated causal eSNPs or in perfect LD. In panel **B**, the proportion of the top SNPs that were the causal eSNPs (or in perfect LD) is shown on the y-axes. For conditional analyses (green), we focused on all top SNPs at each independent locus. For the original eQTL scan (red), we focused on only the best SNP for each significant eGene. We also simulated scenarios where each eGene had three causal eSNPs, and the results were the same.

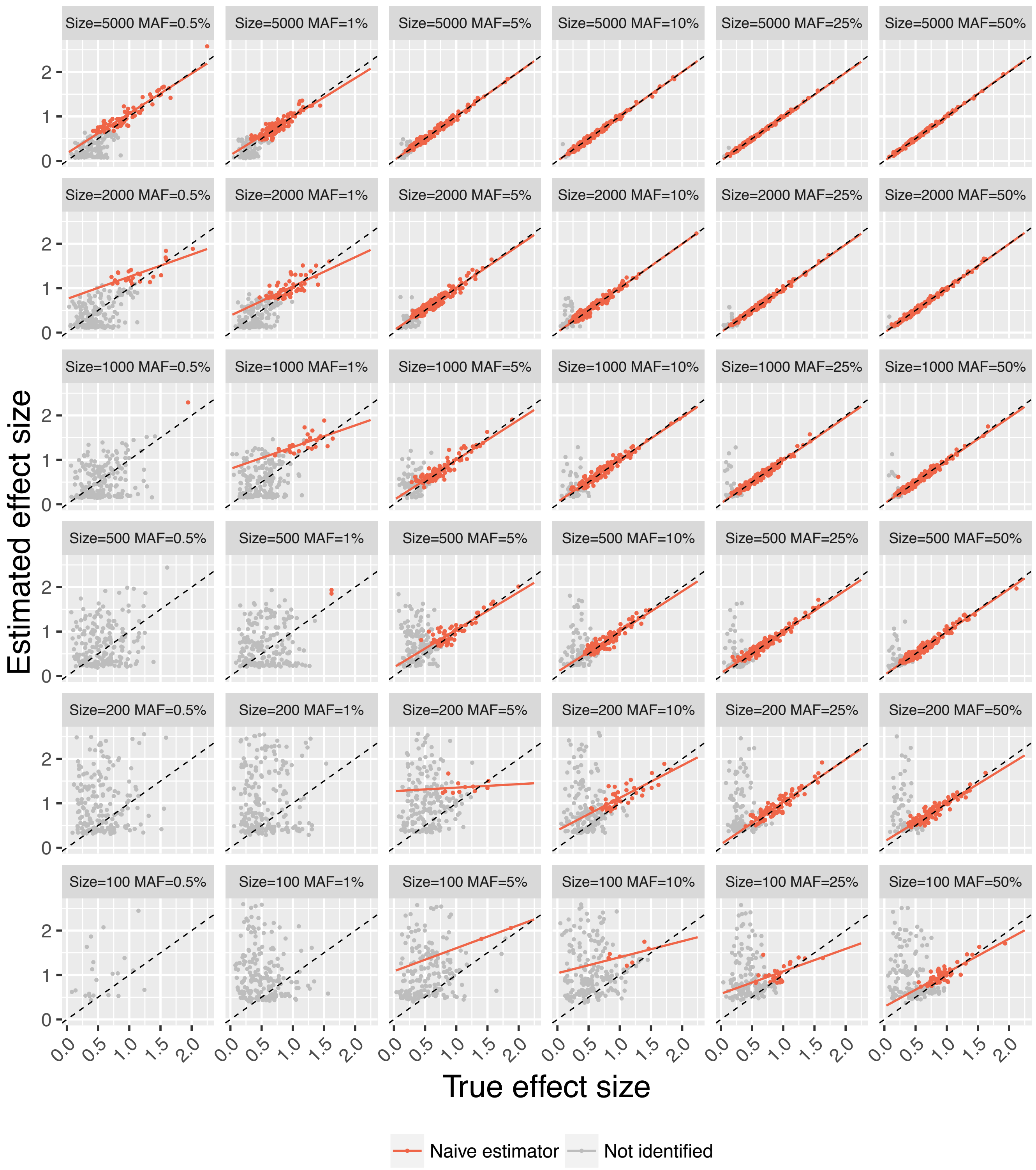

Figure S17. Winner's Curse in eQTL effect size estimation. Each plot represents one scenario, with sample size increasing from bottom to top and minor allele frequency (MAF) of true eSNPs increasing from left to right. Estimated effect sizes are shown on y-axes (naïve estimator from linear regression) and simulated true effect sizes are shown on x-axes. Each dot represents the top SNP of a true simulated eGene. True positive eGenes are shown in red and grey dots are simulated true eGenes that were not significant after hierarchical multiple testing correction using eigenMT for local correction and BH for global correction (eigenMT-BH). Dashed diagonals indicate where the estimated effect size equals to the true value. Red lines are linear regression fit of the naïve estimator on the simulated effect size for the true positive eGenes.

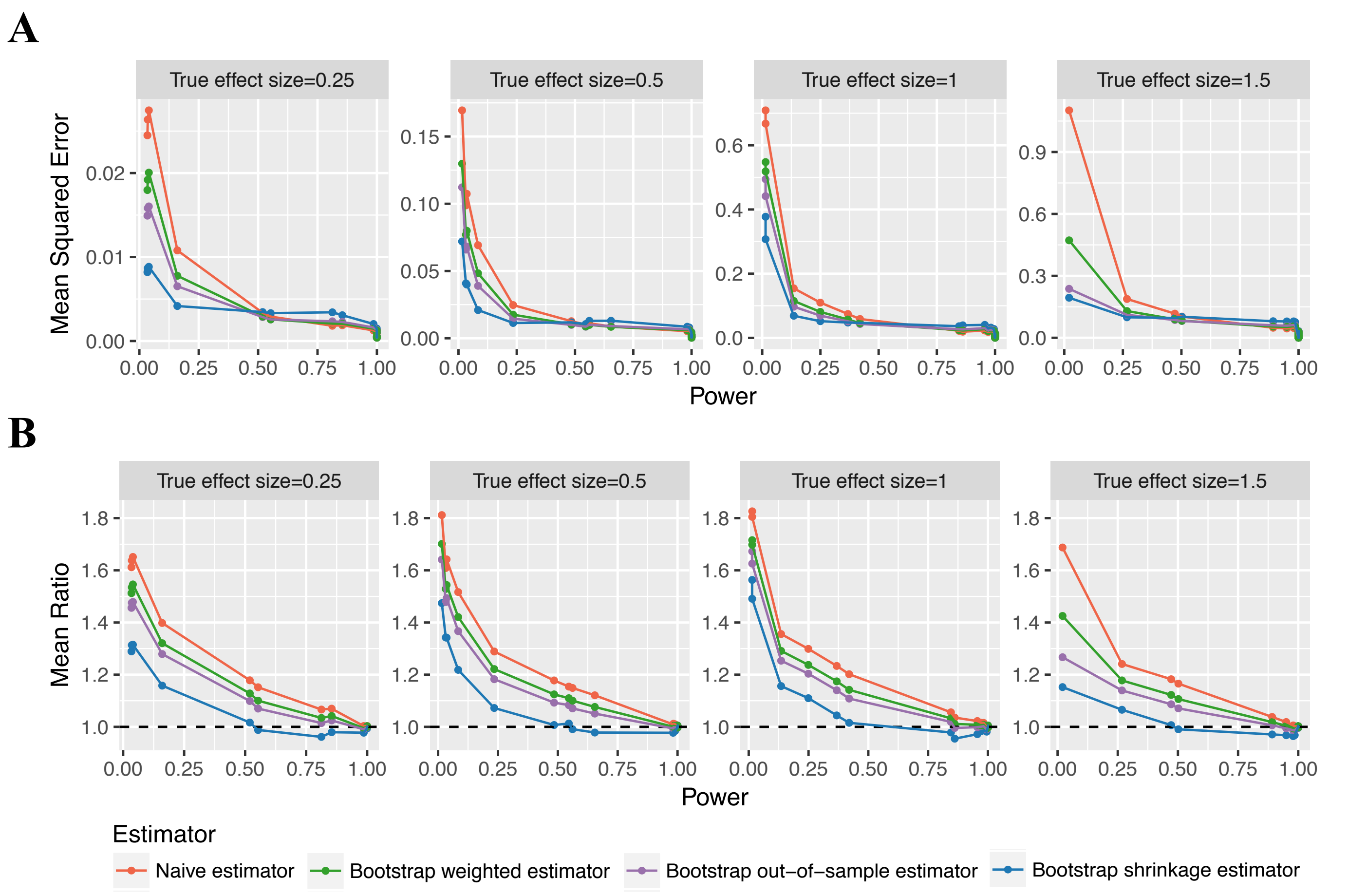

Figure S18. Accuracy of three bootstrap estimators and the naïve estimator. Mean squared error (MSE) with the simulated true effect size (**A**) and the mean ratio of estimated effect size to the simulated true effect size (**B**) were calculated from 10 simulations of scenarios where a constant true effect size (0.25, 0.5, 1, or 1.5 s.d. gene expression per allele) was simulated. Three bootstrap estimators as well as the naïve estimator are shown in different colours. A hierarchical correction procedure using eigenMT for local correction and BH for global correction (eigenMT-BH) was used to correct for multiple testing. The horizontal lines in panel B indicate the ideal unbiased estimator (mean ratio of one).

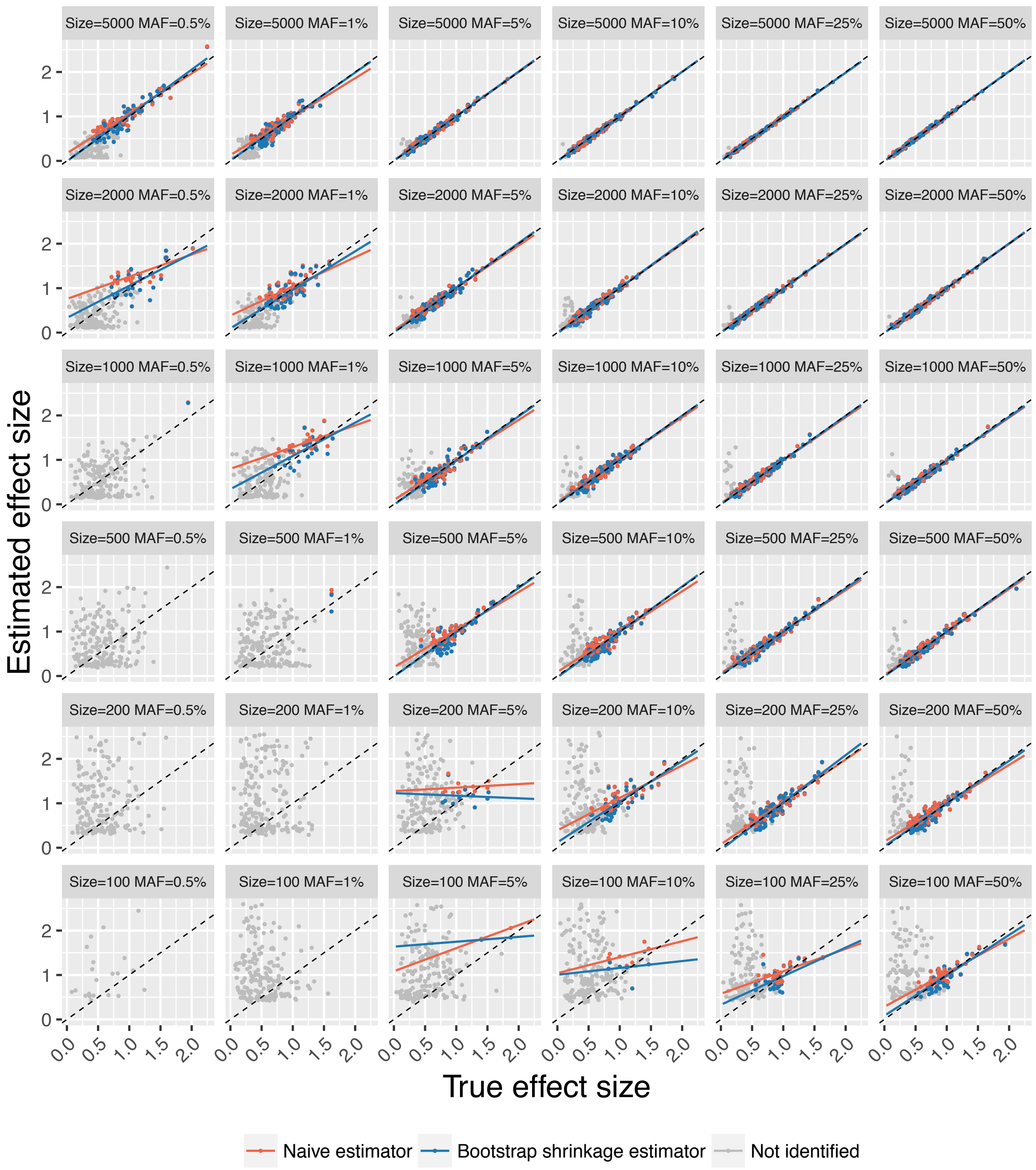

Figure S19. Correction for Winner's Curse by bootstrap method. Each plot represents one scenario, with sample size increasing from bottom to top and minor allele frequency (MAF) of true eSNPs increasing from left to right. Estimated effect sizes are shown on y-axes and true simulated effect sizes are shown on x-axes. Each dot represents the top SNP of a true simulated eGene. True positive eGenes are shown in red (naïve estimator) or blue (bootstrap shrinkage estimator). Grey dots are simulated true eGenes that were not significant after hierarchical multiple testing correction using eigenMT for local correction and BH for global correction (eigenMT-BH). Dashed diagonals indicate where the estimated effect size equals the true simulated effect size. Red (or blue) lines are linear regression fit of the naïve estimator (or bootstrap shrinkage estimator) on the simulated effect size for the true positive eGenes.

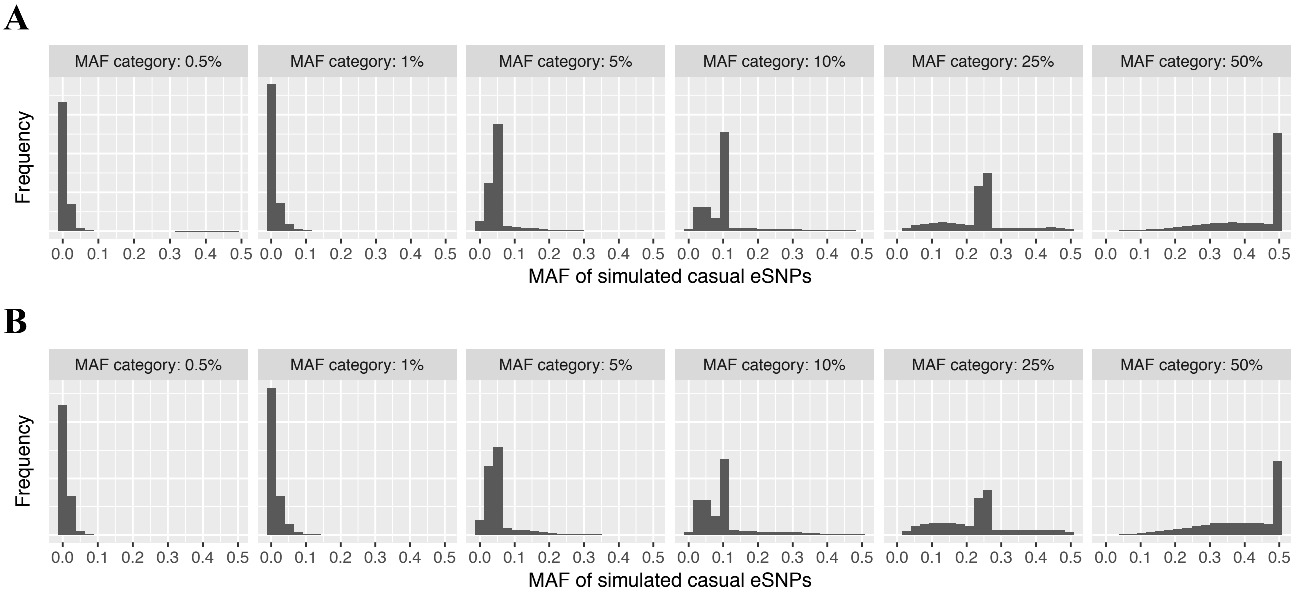

Figure S20. Minor allele frequency (MAF) distribution of causal eSNPs in simulations of two (A) or three (B) causal eSNPs per eGene. Each plot shows the MAF distribution of simulated causal eSNPs in scenarios from a MAF category across all simulated sample sizes. Multiple causal eSNPs for each eGene were selected based on a realistic distribution of linkage disequilibrium, thus the MAFs of the causal eSNPs in one scenario were not always the same.
